# Natural Medicines Targeting Mitochondrial Dysfunction in Spinocerebellar Ataxia Type 3: A Narrative Review of Cellular and Molecular Events

**DOI:** 10.3390/ijms27219623

**Published:** 2026-10-27

**Authors:** Mwafaq Kmail, Noor Asyikin Suaidi, Jin Han Chin, Lee Wei Lim, Kah Hui Wong

**Affiliations:** 1 Department of Anatomy, Faculty of Medicine, Universiti Malaya, Kuala Lumpur 50603, Malaysia; mowafaq.kmail@gmail.com; 2 Department of Forensic Medicine, Sarawak General Hospital, Jalan Hospital, Kuching 93586, Sarawak, Malaysia; asyikin.suaidi@gmail.com; 3 Unit of Biochemistry, Faculty of Medicine, MAHSA University, Jalan SP2, Bandar Saujana Putra, Jenjarom 42610, Selangor, Malaysia; chinjinhan@mahsa.edu.my; 4 Center of Excellence for Brain Health and Neurotechnology, Academy of Life and Natural Sciences, Xi’an Jiaotong-Liverpool University, Suzhou 215123, China; 5 Department of Transfusion Medicine and Clinical Microbiology, Faculty of Allied Health Sciences, Chulalongkorn University, Bangkok 10330, Thailand

**Keywords:** Spinocerebellar ataxia type 3, natural medicines, active compounds, mitochondria

## Abstract

Spinocerebellar ataxia type 3 (SCA3), also known as Machado-Joseph disease, is the most common autosomal dominant spinocerebellar ataxia and is characterized by progressive neurodegeneration for which no Food and Drug Administration (FDA)-approved therapeutics are currently available. Mitochondrial dysfunction has emerged as a central pathogenic mechanism in SCA3, making it a promising therapeutic target. This narrative review aims to evaluate the published evidence on the therapeutic potential of natural medicines and their active compounds targeting mitochondrial dysfunction in SCA3. This review summarizes published evidence on the therapeutic potential of natural medicines and their active compounds targeting mitochondrial dysfunction in SCA3 in preclinical studies. The reviewed studies suggest the therapeutic potential of *Glycyrrhiza inflata*, *Codonopsis pilosula*, *Ginkgo biloba*, *Poria cocos*, *Pueraria lobata*, *Rehmannia glutinosa*, *Curcuma longa*, *Hericium erinaceus*, *Astragalus membranaceus*, *Hemerocallis citrina*, caffeic acid, and resveratrol in targeting mitochondrial dysfunction in SCA3. These natural medicines were reported to regulate multiple mitochondrial-associated pathways, including oxidative stress, mitochondrial membrane potential, mitochondrial bioenergetics, mitochondrial dynamics, mitochondrial quality control, and mitochondria-mediated apoptosis, thereby improving neuronal survival and mitochondrial function in experimental SCA3 models. In conclusion, natural medicines targeting mitochondrial dysfunction represent promising preclinical candidates for further investigation in SCA3. However, further mechanistic investigations, well-designed clinical trials, and optimization of pharmacokinetic properties are required to facilitate their translation into clinical practice.

## 1. Introduction

Spinocerebellar ataxias (SCAs) are autosomal dominant inherited cerebellar disorders [1]. Spinocerebellar ataxia type 3 (SCA3), also known as Machado-Joseph Disease (MJD), is the most common subtype worldwide, accounting for 20–50% of all SCA families and the second most common polyglutamine (polyQ) disease, after Huntington’s disease (HD) [2,3]. The SCA3 ratio varies across populations by geographical location and ethnic background [4]. The worldwide prevalence is estimated to be one to five per 100,000 people [5]. SCA3 is prevalent in many countries, including Brazil, Portugal, China, Germany, the Netherlands, and Japan [6]. As nearly 60% of the world’s population lives in Asia, a large proportion of SCA patients are of Asian origin, with SCA3 being particularly prevalent in China, Thailand, Taiwan, Singapore, and Malaysia [7]. In contrast, SCA3 is less common in the United States, Canada, India, and Australia, and is relatively rare in Italy and South Africa [6]. At present, there is no epidemiological data on SCA in Malaysia, mainly due to the lack of genetic testing facilities and cost. Pooled data from adult Malaysian patients who had undergone genetic testing for SCA at the National university Malaysia Medical Centre and the Institute for Medical Research from 2017 to 2020 revealed that one in 100,000 Malaysians suffers from SCA, with SCA3 being the most common SCA in Malaysia, followed by spinocerebellar ataxia type 3 (SCA2) and spinocerebellar ataxia type 1 (SCA1) [8].

SCA3 is an autosomal dominant neurodegenerative disorder caused by expanded cytosine-adenine-guanine (CAG) repeats in the coding region of ataxin 3 (*ATXN3*) on chromosome 14q32.1, leading to the abnormal production of an elongated polyQ-expanded ataxin-3 [9]. Healthy individuals have up to 44 CAG repeats, whereas SCA3 patients have between 52 and 86 glutamine repeats. An elongated polyQ tract in the C-terminal region of ataxin-3 causes a toxic gain-of-function that induces protein misfolding and aggregation, leading to cytotoxicity and neurodegeneration by disrupting mechanisms of mitochondrial protein quality control [10]. Ataxin-3 is a multifunctional protein involved in various cellular processes, including deubiquitination, cytoskeletal organization, and transcriptional regulation [11]. Accumulation of mutant ataxin-3 aggregates within the nucleus sequesters the 26S proteasome, resulting in protein homeostasis impairment. This disruption of homeostasis causes impaired essential cellular processes, contributes to transcriptional dysregulation, and ultimately drives neuronal cell death [12].

Due to the neurodegenerative process, patients with SCA3 experience significant disabilities and have a shortened lifespan [3]. Individuals diagnosed with SCA3 typically suffer from progressive motor incoordination, progressive ataxia of the body and limbs, dysarthria, hyperreflexia, diplopia, nystagmus, external progressive ophthalmoplegia, and other functional disabilities and neurological manifestations. The symptoms most often appear in adulthood between the second and fifth decades. Therefore, a child is unaware of having SCA3 until signs and symptoms appear in later years. Ambulation difficulty gradually increases, with assistive devices usually required 10 to 15 years after onset, while upper motor neuron lesions are often present and may mimic hereditary spastic paraplegia [13]. SCA3 is the most common genetic disease and was included in the list of rare neurological diseases by the National Rare Disease Committee of the Ministry of Health, Malaysia. With a reduced lifespan, SCA3 is a devastating neurodegenerative disease. The lack of effective therapeutic options remains a major gap in the field.

There is no cure or effective treatment for this fatal, incurable disease to date [14]. Many clinical trials are looking into possible treatments, but there is no Food and Drug Administration (FDA)-approved medicine for SCA3. Targeted therapies for SCA3, e.g., the use of antisense oligonucleotides (ASOs), which are currently recruiting human subjects (ClinicalTrials.gov ID: NCT05822908), identification of biological markers, and preventive interventions for mutation carriers, are currently realistic options [15]. The trials focus on removing harmful protein buildup. Neuromotor exercise training and physical therapy targeting on coordination and balance have been shown to halt the progression of SCA3 [3,16]. There is a necessity to develop therapeutics that delay the progression of SCA3, a need that is shared by almost all movement disorders.

For centuries, traditional medicines have supported human health across cultures and civilizations. Today, as the global healthcare landscape evolves, these age-old remedies are gaining renewed relevance, not as alternatives, but as valuable complements to modern medicines. Mitochondria, with their intricate networks of functions and information processing, are crucial in health regulation and disease progression. Particularly, mitochondrial dysfunctions are identified in many common pathologies, including neurodegeneration, cardiovascular diseases, metabolic syndrome, and cancer. Although the potential roles of natural medicines have been investigated for SCA3 [9,17,18,19,20], the understanding of in-depth and fundamental mechanisms targeting mitochondrial dysfunction remains elusive. Therefore, the narrative review aims to critically elucidate the current understanding of natural medicines targeting mitochondrial dysfunction in SCA3. We examined a total of seven preclinical studies encompassing twelve natural medicines, which were tested on five transgenic cell lines and animal models of SCA3. As far as mitochondrial dysfunction is concerned, natural medicines exert their therapeutic effects in SCA3 through attenuation of mitochondrial oxidative stress, enhancement of mitochondrial biogenesis and bioenergetics, preservation of mitochondrial membrane potential and mitochondrial integrity, and suppression of mitochondria-mediated apoptosis. It is foreseen that these findings may advance the efficiency and effectiveness of natural medicines for the management of SCA3.

## 2. Pathogenesis of Spinocerebellar Ataxia Type 3

**Figure 1:**
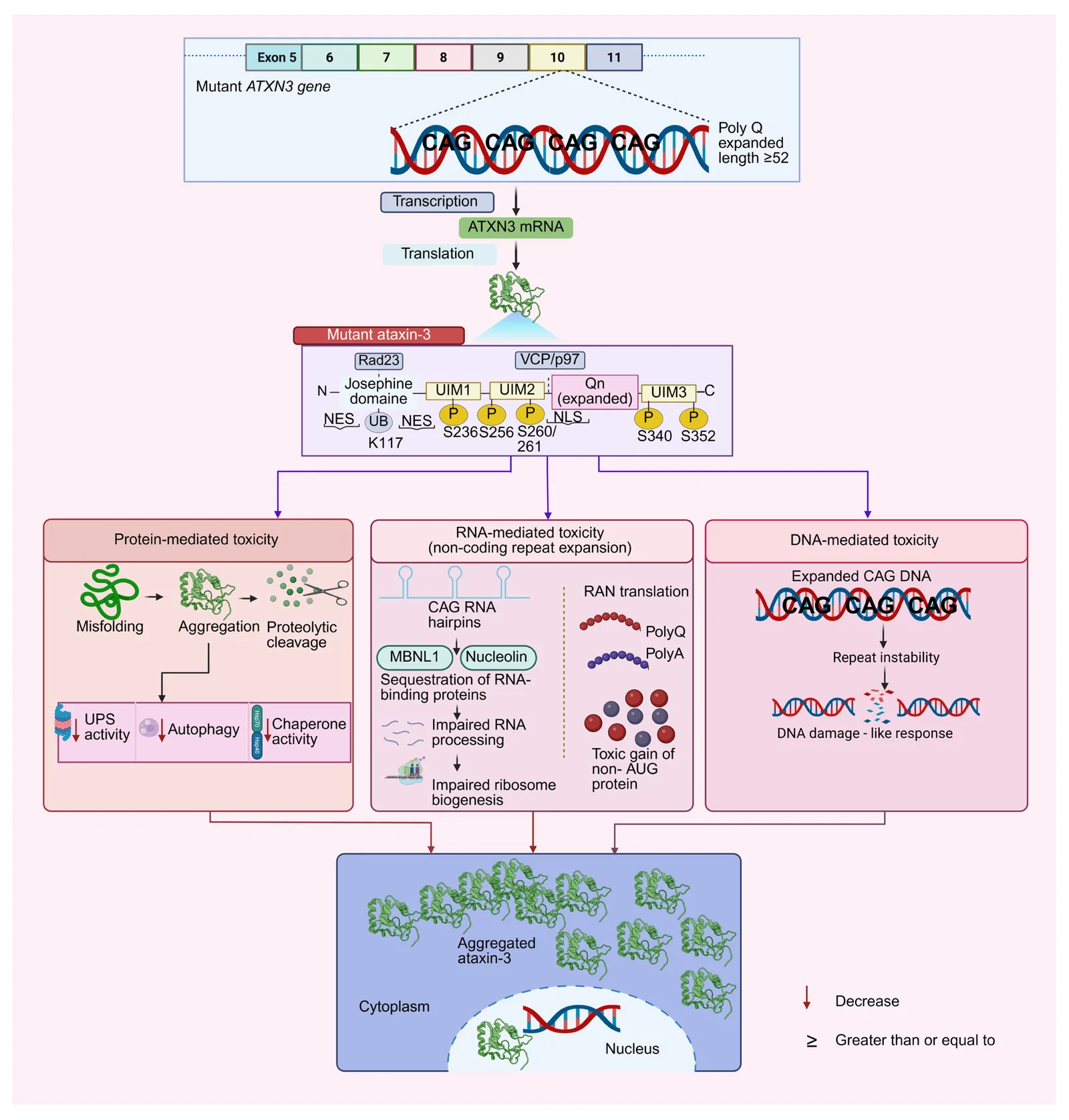
Pathogenic mechanisms of mutant ataxin-3. Expanded polyQ repeats ≥52 drives post-translational modifications and domain interactions (Rad23, VCP/p97). Toxicity occurs via two main pathways: (i) Protein toxicity, where misfolding, aggregation, and cleavage impair the UPS, autophagy, and chaperones (Hsp70/40); and (ii) RNA toxicity, where CAG hairpins sequester RNA-binding proteins (MBNL1, nucleolin) and RAN translation of non-toxic AUG proteins (polyQ, polyA). Both pathways drive nuclear and cytoplasmic accumulation of aggregated ataxin-3. AUG, adenine-uracil-guanine; CAG, cytosine-adenine-guanine; DNA, deoxyribonucleic acid; Hsp40/70, heat shock protein 40/70; MBNL1, muscleblind-like splicing regulator 1; NES, nuclear export signal; NLS, nuclear localization signal; N, N-terminal; P, phosphorylation; polyA/polyQ, polyalanine/polyglutamine; RAN, repeat-associated non-AUG; S236, S256, S260/261, S340, S352, serine phosphorylation sites at their respective positions; RNA, ribonucleic acid; UB, ubiquitin; UIM, ubiquitin-interacting motif; UPS, ubiquitin–proteasome system; VCP, valosin-containing protein. The schematic diagram was created using BioRender.com.

According to genetic nomenclature, identified SCAs are numbered based on the order in which their genetic loci were discovered. Additionally, SCAs are classified into two groups based on their underlying mutation types, i.e., repeat expansions and point mutations [21]. SCA3 is an autosomal dominant disease caused by the abnormal expansion of CAG repeats in the *ATXN3* [22]. Figure 1 shows the pathogenic mechanisms of mutant *ATXN3*. SCA3, like other polyQ diseases from CAG repeat expansions, may share similar pathogenic mechanisms. Investigating the genotype–phenotype relationship helps elucidate pathogenesis and develop effective therapies [21,23].

The pathogenic basis of SCA3 is primarily driven by a CAG repeat expansion located in exon 10 of the *ATXN3* on chromosome 14q32.1 [24]. The expanded CAG tract is transcribed into an *ATXN3* messenger ribonucleic acid (mRNA) containing an elongated CAG repeat sequence and is translated into an ataxin-3 protein with an abnormally expanded polyQ tract. Thus, the genetic mutation directly links the expanded CAG sequence in *ATXN3* to the structural alteration of the ataxin-3 protein. The length of the CAG repeat is inversely correlated with disease onset, with larger expansions related to earlier and more severe clinical presentation. Ataxin-3 is widely expressed and can move between the nucleus and cytoplasm, where it participates in the ubiquitin–proteasome system, leading to ribonucleic acid (RNA) toxicity [25]. Ataxin-3 is widely expressed and can move between the nucleus and cytoplasm, where it participates in the ubiquitin–proteasome system. In SCA3, mutant ataxin-3 accumulates in neurons and forms toxic aggregates that disrupt protein degradation and impair cellular homeostasis, leading to progressive neuronal dysfunction [10].

### 2.1. PolyQ-Expanded Ataxin-3 Protein

The ataxin-3 is a 361-amino-acid protein composed of an N-terminal Josephin domain (JD) responsible for deubiquitinating activity and a flexible C-terminal region containing multiple regulatory motifs involved in protein quality control. The JD harbours a catalytic triad that enables cleavage of ubiquitin chains, conferring deubiquitinating enzyme activity. At the same time, the C-terminal region includes ubiquitin-binding site 1 and ubiquitin-binding site 2, which mediate interactions with ubiquitin and proteasome-associated factors such as Rad23 [26,27]. In addition, the C-terminal region contains three ubiquitin-interacting motifs that bind polyubiquitin chains, as well as a p97-binding motif that links ataxin-3 to the p97 protein quality control machinery, further integrating ataxin-3 into the ubiquitin–proteasome system [26].

The C-terminus also includes a variable-length polyQ tract encoded by CAG repeats, where expansion leads to the pathogenic *ATXN3*-polyQ form. Additionally, ataxin-3 contains nuclear localization and export signals that regulate its subcellular distribution [28]. In the brain, several *ATXN3* splice variants exist, but only a few are translated into functional proteins. The predominant brain isoform contains 11 exons [10]. Ataxin-3 is involved in multiple cellular processes, including the ubiquitin–proteasome system, aggresome formation, cytoskeletal organization, deoxyribonucleic acid (DNA) binding, and interactions with transcription factors [3]. However, ataxin-3 is generally considered non-essential, as studies using knockout models in *Caenorhabditis elegans* and C57BL/6 mice showed this model does not produce phenotypic abnormalities [27,29,30].

The translation of expanded CAG repeats results in the production of mutant ataxin-3 containing an abnormally elongated PolyQ tract, causing SCA3. In healthy conditions, ataxin-3 is predominantly localized in the cytoplasm, whereas the polyQ-expanded form is present in both the cytoplasm and nucleus and progressively forms intranuclear aggregates in specific neuronal populations [31]. When the polyQ tract expands beyond a normal threshold, it becomes an amyloidogenic structure that promotes protein misfolding and aggregation, leading to the formation of toxic intracellular inclusions and ultimately contributing to neurodegeneration. In addition, polyQ-expanded proteins can disrupt cellular proteostasis by sequestering key co-chaperones such as heat shock protein J1 into aggregates, thereby impairing protein quality control and promoting further accumulation of misfolded ataxin-3 [32]. Mutant ataxin-3 undergoes proteolytic cleavage to generate shorter polyQ-containing fragments with increased aggregation propensity and cytotoxicity. This process is primarily mediated by calpain proteases and is enhanced in neurons due to elevated calcium influx, contributing to their selective vulnerability in SCA3 [10].

### 2.2. Noncoding Repeat Expansions in ATXN3

Beyond protein-mediated toxicity, the expanded CAG repeat in *ATXN3* also contributes to SCA3 pathogenesis through effects at the DNA and RNA levels, which increase the pathogenesis complexity [10,33]. A study by Li et al. using *Drosophila melanogaster* showed that expanded CAG repeat RNA alone can induce neuronal toxicity, even without protein production [33]. Another study by Wang et al. using *C. elegans* and C57BL/6 mice showed that the RNA toxicity from CAG depends on a length-dependent manner; expression of 83 CAG repeats did not produce an observable phenotype, whereas 200 repeats resulted in severe toxicity, including premature death [34]. Expanded CAG repeat RNA adopts abnormal secondary structures, such as hairpins, which can sequester RNA-binding proteins like muscleblind-like splicing regulator 1 (MBNL1), leading to dysregulation of RNA processing and cellular dysfunction [10].

Additionally, expanded CAG repeats can trigger repeat-associated non-AUG (RAN) translation, producing toxic proteins from both sense and antisense strands without a canonical start codon [35]. Jazurek-Ciesiolka et al. showed that expanded CAG repeats within the *ATXN3* sequence context undergo RAN translation, leading to the production of toxic polyQ and polyalanine (polyA) proteins, which cause cellular toxicity through nuclear impairment and apoptosis [36].

Recent studies further highlight this phenomenon, showing that expanded CAG repeats can create toxic sites within the genome that, through interactions with normal DNA metabolic processes, may ultimately lead to cell death [37,38]. CAG repeats can lead to DNA repeat instability, especially when both sense and antisense transcription occur, resulting in increased repeat expansions and contractions. This instability triggers a DNA damage-like response [39].

## 3. Consequences of Ataxin-3 Misfolding

### 3.1. Dysregulation of Transcription

Studies have shown that removal of the *ATXN3* does not produce obvious neurodegenerative effects, suggesting that SCA3 is primarily driven by a dominant gain-of-function mechanism rather than loss of normal *ATXN3* function [27,29,30]. Many polyQ-expanded proteins are involved in transcriptional regulation, and their mutation can disrupt normal gene expression patterns in the nervous system. Therefore, mutant *ATXN3* likely contributes to disease by altering transcriptional processes [40]. Ataxin-3 has multiple roles, including regulating transcription and maintaining genomic stability following DNA damage [28]. Chromatin, a DNA-protein structure in the nucleus, controls DNA accessibility and thereby regulates transcription and DNA replication. Under normal conditions, ataxin-3 promotes chromatin compaction by recruiting histone deacetylase 3 (HDAC3) to chromatin, leading to gene repression of matrix metallopeptidase 2 (*MMP2*). In contrast, mutant ataxin-3 sequesters histone deacetylase 3 (HDAC3) and prevents its proper localization, resulting in a more open chromatin state, increased transcription, and altered replication [28,41].

Additionally, polyQ-expanded ataxin-3 impairs interactions with coactivators such as CREB-binding protein (CBP), p300, and p300/CBP-associated factor (PCAF), reducing their activity. These changes lead to downregulation of genes involved in neurotransmission and signaling pathways, alongside upregulation of inflammatory and apoptosis-related genes, ultimately contributing to neuronal dysfunction in SCA3 [40,42]. These expanded RNAs can drive neuronal dysfunction, with toxicity depending on repeat length and RNA structure. RNA-binding proteins such as MBNL1 can further enhance this toxicity by increasing CAG RNA levels and promoting degeneration [33].

### 3.2. RNA Toxicity

Mutant *ATXN3* leads to toxic repeat-containing RNA species that independently contribute to neurodegeneration [10]. Expanded CAG RNAs can sequester nucleolin, a key regulator of rRNA transcription, leading to reduced ribosome biogenesis and activation of the ribosomal protein(RP)–MDM2–p53 pathway, ultimately triggering apoptosis [43,44,45]. Additionally, these toxic RNAs may disrupt gene transcription by sequestering transcription factors and altering signaling pathways such as protein kinase B (Akt)/glycogen synthase kinase-3 beta (GSK3-β) [43]. SCA3 has also been associated with dysregulation of RNA-binding proteins involved in RNA processing and stress granule formation, including fused in sarcoma (FUS), TAR DNA-binding protein 43 (TDP-43), and ataxin-2, which are found in pathological aggregates and linked to altered RNA homeostasis [46].

### 3.3. Glutamate Dysregulation

Glutamate is an amino acid and the primary excitatory neurotransmitter in the nervous system, playing essential roles in synaptic transmission, neuronal plasticity, learning, and memory [47]. In addition, it is required for the synthesis of glutathione, a key antioxidant involved in maintaining cellular redox balance [48]. Glutamate mediates its effects by binding to specific receptor proteins on the neuronal cell surface, and therefore glutamatergic neurotransmission is tightly regulated through controlled synaptic release and rapid uptake rather than extracellular enzymatic degradation [49]. Under physiological conditions, extracellular glutamate levels are maintained by sodium ion (Na^+^)-dependent excitatory amino acid transporters (EAATs), which are highly expressed in astrocytes and are responsible for the rapid clearance of glutamate from the synaptic cleft to prevent excessive receptor activation [50,51]. Impairment of EAAT function can therefore lead to glutamate accumulation, prolonged receptor stimulation, and increased risk of excitotoxic neuronal injury.

Excessive activation of glutamate receptors results in increased intracellular calcium ion (Ca^2+^) influx, triggering oxidative stress, mitochondrial dysfunction, and activation of cell death pathways, ultimately leading to neuronal injury, a process known as glutamate excitotoxicity [52,53,54]. Evidence of glutamatergic dysregulation has also been reported in SCA3-related models. In ataxin-3-Q79 transgenic mice, reduced expression of vesicular glutamate transporter 2 (VGLUT2) and synaptotagmin 1 (SYT1) in the cerebellum suggests impaired Ca^2+^-dependent glutamate release from glutamatergic neurons [42]. Furthermore, in vitro studies using SCA2- and SCA3-induced pluripotent stem cell (iPSC)-derived neurons demonstrated that glutamate exposure exacerbates excitotoxic vulnerability, evidenced by dysregulation of glutamate receptor- and transporter-related genes (glutamate ionotropic receptor AMPA type subunit 1, *GRIA1*, glutamate ionotropic receptor AMPA type subunit 2, *GRIA2*, glutamate ionotropic receptor AMPA type subunit 4, *GRIA*4, glutamate metabotropic receptor 3, *GRM3*, and solute carrier family 17 member 6, *SLC17A6*), increased intracellular Ca^2+^ levels, and impaired mitochondrial function. Collectively, these findings indicate that SCA3 neurons exhibit heightened sensitivity to glutamate-mediated excitotoxic stress, which may contribute to neurodegeneration [55].

Collectively, ataxin-3 misfolding leads to dysregulation of transcription, RNA toxicity, and glutamate imbalance, contributing to cellular stress and neuronal injury in SCA3 [21]. These mechanisms can drive or amplify mitochondrial dysfunction through intersecting pathways, rather than operating as fully independent mechanisms [21,55]. Importantly, current evidence does not establish mitochondrial dysfunction as the initiating cause of SCA3 pathogenesis. However, mitochondrial abnormalities may arise as a consequence of ataxin-3 misfolding, therefore amplifying neuronal injury [14,56]. Mitochondrial dysfunction should be considered as a potential contributor and amplifier of SCA3 pathogenesis, rather than the primary genetic driver.

## 4. Impairment of Mitochondrial Morphology, Function and Homeostasis

### 4.1. Mitochondrial Morphology

Ataxin-3 misfolding in SCA3 contributes to cellular dysfunction, particularly through toxic C-terminal fragments [14]. This organelle targeting represents a secondary pathological mechanism rather than the initiating genetic cause of SCA3. Mutant ataxin-3 exhibits increased interaction with mitochondrial proteins, supporting a direct link to mitochondrial impairment [14,57]. In addition, polyQ aggregates have been reported to accumulate within mitochondria, further disrupting mitochondrial integrity [19]. In healthy cells, mitochondrial fission and fusion are essential dynamic processes that maintain mitochondrial integrity by facilitating the repair of damaged or aged mitochondria, regulating mitochondrial abundance, and mediating the removal of dysfunctional organelles through a unique process called mitophagy [58,59]. In SCA3, however, these processes become dysregulated. Truncated ataxin-3 has been shown to promote mitochondrial fragmentation by enhancing fission and suppressing fusion through downregulation of MFN1 (mitofusin 1) and MFN2 (mitofusin 2), resulting in loss of mitochondrial membrane potential, increased oxidative stress, and dysregulation of antioxidant defense mechanisms [14,60].

Consistent with these mechanisms, SCA3 patient-derived fibroblasts and mouse embryonic fibroblasts expressing mutant ataxin-3 with 148 CAG repeats display prominent abnormalities of mitochondrial morphology. The cells exhibit fragmented and ring-shaped mitochondria, indicating disrupted mitochondrial network integrity. Additionally, mouse embryonic fibroblasts show mitochondrial condensation with prominent perinuclear clustering. These alterations are associated with reduced long-form optic atrophy 1 (L-OPA1) and MFN2 expression, suggesting impaired mitochondrial fusion in SCA3 [61].

Further supporting mitochondrial structural disruption, in vitro studies using mouse embryonic fibroblasts expressing mutant ataxin-3 with 148 CAG repeats have demonstrated fragmented, poorly interconnected mitochondria with reduced branching and length, reflected by decreased form factor and aspect ratio. These changes are accompanied by disrupted cristae organization, leading to reduced mitochondrial respiratory capacity and increased susceptibility to apoptosis. Disruption of cristae architecture may further impair respiratory chain efficiency and adenosine triphosphate (ATP) production [61,62]. Mitochondrial fragmentation is also associated with increased pro-fission factor, dynamin-related protein 1 (Drp1) regulated by calcium-dependent calcineurin signaling [63].

### 4.2. Mitochondrial Function

Evidence from multiple experimental systems indicates that mitochondrial dysfunction is a central feature of SCA3 pathogenesis. The polyQ-expanded ataxin-3 has been reported to accumulate within mitochondria, where it can disrupt mitochondrial integrity and impair mitophagy, thereby compromising mitochondrial quality control [19]. In parallel, mitochondrial dysfunction is known to enhance reactive oxygen species (ROS) production, further amplifying oxidative stress and cellular injury [64].

Evidence from in vivo studies further supports early mitochondrial involvement in disease progression. In SCA3 transgenic mouse models, Kazachkova et al. demonstrated significant mitochondrial DNA (mtDNA) depletion in affected tissues, detectable before clinical symptoms. As mtDNA encodes essential components of the oxidative phosphorylation system, its reduction is expected to impair mitochondrial bioenergetics and contribute to disease onset and progression [65]. Furthermore, SCA3 patient-derived cellular models also reveal mitochondrial bioenergetic defects. Harmuth et al. showed that fibroblasts expressing polyQ-expanded ataxin-3 exhibit reduced ATP production and decreased expression of key oxidative phosphorylation proteins, particularly succinate dehydrogenase (SDHB) and ubiquinol-cytochrome c reductase core protein 2 (UQCRC2), indicating impaired respiratory chain function and mitochondrial bioenergetic failure [61]. In addition, proteomic interaction studies demonstrate that mutant ataxin-3 shows reduced association with multiple mitochondrial and metabolic proteins, including components of complex I (NADH-dehydrogenase, NDU, alpha subcomplex, F, assembly factor, AF), complex IV (cytochrome c oxidase subunit 6B1, COX6B1, cytochrome c oxidase subunit 7C, COX7C, mitochondrially encoded cytochrome c oxidase II, MT-CO2), ATP synthase (ATP synthase F(0) complex subunit k, mitochondrial, ATP5K), and enzymes involved in intermediary metabolism such as pyruvate dehydrogenase E1 component subunit beta (PDHB), ATP citrate lyase (ACLY), and hydroxyacyl-CoA dehydrogenase trifunctional multienzyme complex subunit beta (HADHB), collectively indicating widespread disruption of mitochondrial energy metabolism in SCA3 [66].

Mitochondrial dysfunction is also associated with altered mitochondrial dynamics, including an imbalance between fission and fusion, which may impair mitochondrial integrity and mitophagy, thereby compromising mitochondrial quality control [67].

At the level of neuronal apoptosis, expression of murine ataxin-3 containing 79 CAG repeats (ATXN3-79Q) in primary central nervous system (CNS) neurons triggers pronounced mitochondrial dysfunction leading to mitochondrial outer membrane permeabilization and release of cytochrome c (cyt c) and second mitochondria-derived activator of caspases (Smac) into the cytosol. This activates the intrinsic apoptotic cascade via caspase-9 and caspase-3. These events are accompanied by increased Bcl-2-associated X protein (Bax) and decreased B-cell lymphoma-extra-large (Bcl-Xl), promoting mitochondrial destabilization and neuronal loss [68]. Collectively, these findings position mitochondrial integrity, bioenergetics, and apoptotic signaling as interconnected and central contributors to SCA3 neurodegeneration.

### 4.3. Mitochondrial Homeostasis

Mitochondrial homeostasis depends on efficient mitochondrial quality control mechanisms, particularly mitophagy, which removes damaged mitochondria and maintains a healthy mitochondrial network. In SCA3, polyQ-expanded ataxin-3 disrupts these processes by accumulating within mitochondria and impairing mitochondrial quality control [19]. Defective deubiquitination of voltage-dependent anion channel 1 (VDAC1) and reduced Parkin recruitment further compromise mitophagy [61]. The PTEN-induced kinase1 (PINK1)/Parkin pathway is a major regulator of mitochondrial quality control. Under normal conditions, mitochondrial damage promotes PINK1 accumulation on the outer mitochondrial membrane, leading to Parkin activation and mitophagy initiation. However, in SCA3 models, phosphorylation of parkin at serine 65 (Ser65) remains unchanged, suggesting impaired activation of the PINK1/Parkin pathway [59,69]. Moreover, ataxin-3 directly interacts with Parkin, and the polyQ-expanded protein enhances Parkin deubiquitination, reducing Parkin stability and promoting its autophagic degradation [70]. Despite reduced Parkin levels, evidence of mitochondrial loss indicates that mitophagy is altered rather than completely suppressed. Consequently, mitochondrial clearance may shift toward Parkin-independent pathways involving Bcl-2 interacting protein 3 (BNIP3), Nip3-like protein X (NIX), and BCL2-like 13 (BCL2L13) [59].

Evidence from cellular models further supports impaired mitochondrial homeostasis. In mouse embryonic fibroblasts expressing mutant ataxin-3 with 148 CAG repeats, disrupted autophagy-related genes and proteins are initially upregulated, indicating an early compensatory response. However, this response is not maintained over time, as autophagy markers return to baseline while pro-apoptotic signaling increases. Under endoplasmic reticulum (ER) stress, mutant cells exhibit impaired autophagy, persistent ER stress, and ER swelling, whereas wild-type cells restore cellular homeostasis through an effective autophagic response [62].

Complementary evidence further demonstrates disrupted mitochondrial homeostasis in vivo. In the Ki150 SCA3 knock-in mice with 150 CAG repeats, mutant ataxin-3 shows reduced interactions with proteins involved in oxidative phosphorylation, mitochondrial dynamics, and mitochondrial transport, including NADH:ubiquinone oxidoreductase subunit A5 (NDUFA5), NADH:ubiquinone oxidoreductase subunit A8 (NDUFA8), NADH:ubiquinone oxidoreductase subunit A10 (NDUFA10), NADH:ubiquinone oxidoreductase subunit A12 (NDUFA12), NADH:ubiquinone oxidoreductase subunit S4 (NDUFS4), COX6B1, COX7C, ATP5K, voltage-dependent anion channel 3 (VDAC3), optic atrophy 1 (OPA1), ganglioside-induced differentiation-associated protein 1 (GDAP1), leucine zipper-EF-hand-containing transmembrane protein1 (LETM1), and vacuolar protein sorting ortholog 35 (VPS35). These molecular alterations are accompanied by significantly reduced mitochondrial motility and slower anterograde and retrograde transport in cerebellar neurons, indicating impaired mitochondrial maintenance and trafficking [66]. Overall, evidence from cellular and animal models suggests that mutant ataxin-3 impairs mitochondrial quality control by disrupting mitophagy pathways, compromising adaptive autophagic responses, and altering mitochondrial transport dynamics. These defects collectively contribute to the loss of mitochondrial homeostasis observed in SCA3.

**Figure 2:**
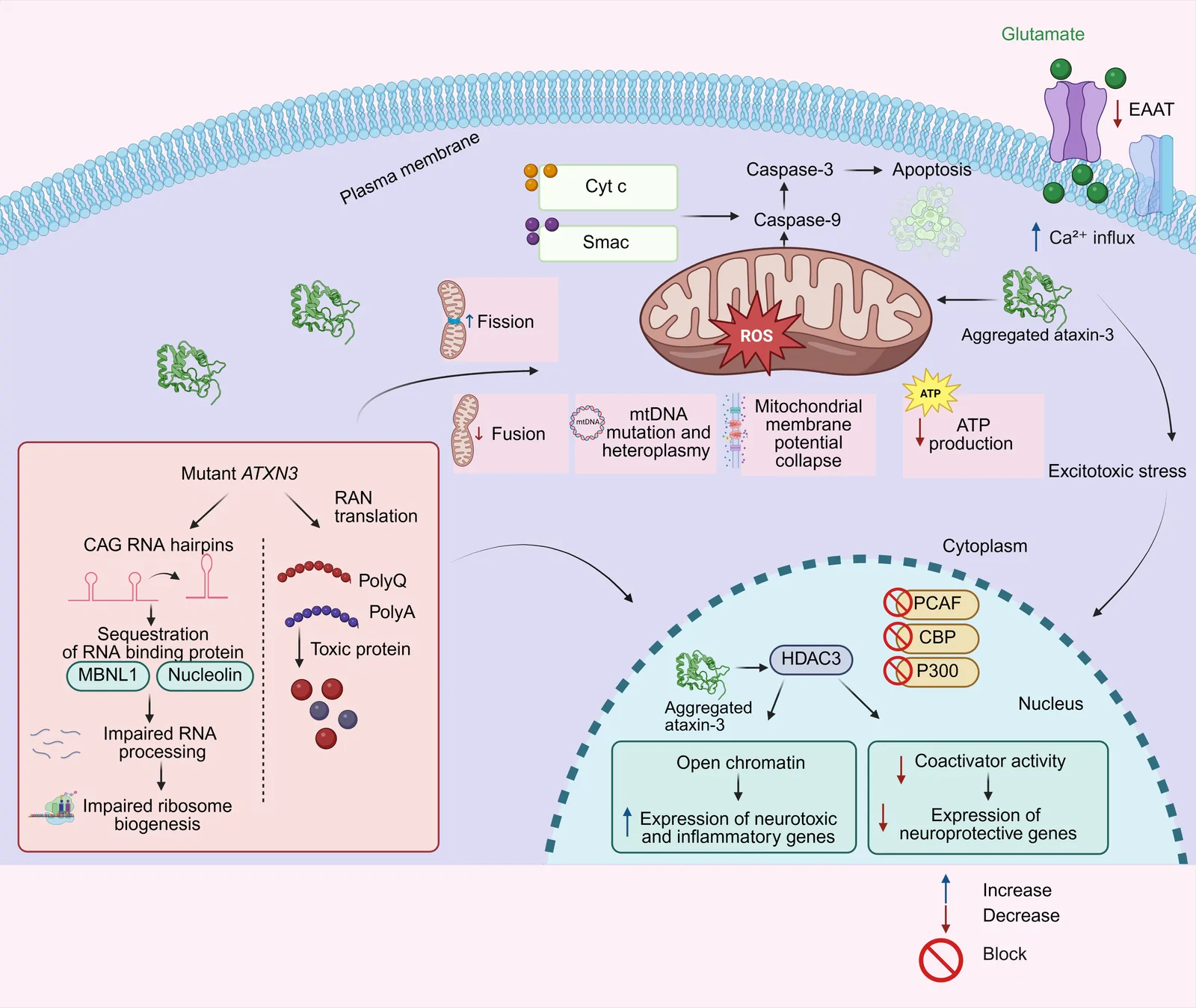
Mitochondrial dysfunction and apoptotic pathways in SCA3, highlighting the central role of mitochondrial decay under cellular stress. Glutamate excitotoxicity and toxic proteins (polyQ/polyA) drive mitochondrial membrane potential collapse, altered fission dynamics, and decreased ATP production. The resulting accumulation of ROS triggers the release of cyt c and Smac, activating caspase-9 and caspase-3 to initiate apoptosis. ATP, adenosine triphosphate; ATXN3, ataxin-3; Ca^2+,^ calcium ion; CAG, cytosine-adenine-guanine; CBP, CREB-binding protein; cyt c, cytochrome c; EAAT, excitatory amino acid transporter; HDAC3, histone deacetylase 3; MBNL1, muscleblind-like splicing regulator 1; mtDNA, mitochondrial DNA; P300, histone acetyltransferase p300; PCAF, p300/CBP-associated factor; PolyA, polyalanine; PolyQ, polyglutamine; ROS, reactive oxygen species; Smac, second mitochondria-derived activator of caspases. The schematic diagram was created using BioRender.com.

Figure 2 highlights the impact of aggregated ataxin-3 on mitochondrial dysfunction and ROS accumulation, and the subsequent apoptotic pathway.

Collectively, these findings position bioenergetic failure, ROS accumulation and oxidative stress, altered mitochondrial dynamics, impaired mitophagy, and accelerated mitochondrial apoptotic signaling as interconnected and central contributors to SCA3 neurodegeneration.

## 5. Treatment Modalities of SCA3

Currently, clinical management of SCA3 remains supportive, primarily to alleviate symptoms and preserve motor and functional abilities rather than to modify disease progression [21]. Pharmacological and non-pharmacological approaches focus on improving quality of life through management of symptoms, use of botulinum toxin for dystonia or spasticity, and multidisciplinary rehabilitation, including physical, occupational, and speech therapy [10,71]. These interventions provide functional benefit but do not affect the underlying polyQ-mediated neurodegenerative process.

To date, no therapeutics have been approved to slow or halt neurodegeneration in SCA3 [72]. Nevertheless, some clinical trials targeting disease-relevant pathways have revealed encouraging results [71]. These include modulation of glutamatergic transmission, such as troriluzole and VO659 (an ASO) to the target RNA, and modulating protein expression [5,73], and serotonergic modulation approaches such as NLX-112 (befiradol/F13640) [74]. Beyond neurotransmitter-based approaches, emerging therapies aim to target key pathogenic mechanisms, including cerebellar circuit dysfunction, transcriptional dysregulation, mitochondrial impairment, and disrupted proteostasis, all of which contribute to progressive neuronal loss [21]. Despite this expanding therapeutic landscape, the FDA has yet to approve any of the drugs [72].

### Therapeutic Development for SCA3

Therapeutic development for SCA3 has progressively shifted from symptomatic management toward innovative, multi-target therapeutic strategies that can effectively halt the progression of SCA3 [75]. According to the National Ataxia Foundation, therapeutic agents developed for SCA3 are formulated based on their pathogenic mechanism, such as glutamate regulation, serotonin regulation, and *ATXN3* suppression.

The NLX-112, developed by Neurolixis Incorporated, is a centrally acting, highly potent, and selective full agonist of serotonin 1A (5-HT_1A_) receptors, and is currently considered a preclinical candidate for SCA3 [76]. Furthermore, in vivo studies using a *C. elegans* model showed that treatment with 10 μM befiradol improved motor function, increased locomotor velocity, and reduced mutant ataxin-3 aggregation [77]. However, these findings are limited by the relatively simple nervous system of *C. elegans*, which reduces translational relevance to mammalian neurobiology, as well as the lack of validation in mammalian disease models and the absence of long-term safety and efficacy data. In a newly published results by bioRxiv, using a mouse model, NLX-112 has been shown to improve motor coordination and balance with prolonged administration (0.625 and 5 mg/kg/day for 34 weeks) started before the onset of the symptoms [78]. Additionally, a Phase 2a clinical trial (ClinicalTrials.gov ID: NCT05148884) in Parkinson’s disease patients demonstrated that NLX-112 was generally safe and well tolerated over an 8-week treatment period [76]. However, this study was not conducted in SCA3 patients and primarily assessed short-term safety rather than disease-modifying efficacy. Therefore, although early findings are encouraging, further studies in SCA3-specific models and patient populations are required to establish its therapeutic potential.

The VO659, developed by Vico Therapeutics B.V., is currently under investigation in a Phase 1/2a clinical trial (ClinicalTrials.gov ID: NCT05822908) for the treatment of SCA1, SCA3, and Huntington’s disease. The compound is administered via intrathecal injection across multiple dose levels in an estimated total of 68 participants [79]. Key limitations of this study include the small sample size, absence of a control group, and inclusion of heterogeneous polyQ disorders, which may limit interpretation of efficacy signals and generalizability of outcomes. No clinical results have been reported to date.

Glutamate dysregulation has also been explored as a therapeutic target in SCA3. Riluzole has been investigated in a transgenic SCA3 Q70 mouse model, where it reduced soluble ataxin-3 levels; however, it did not improve motor deficits, body weight, or behavioral abnormalities. In addition, riluzole treatment was associated with reduced calbindin expression in Purkinje cells, suggesting potential cerebellar dysfunction [80]. BHV-4157 (troriluzole), developed by Biohaven Pharmaceuticals Incorporated, is a third-generation tripeptide prodrug of riluzole designed to improve bioavailability, pharmacokinetics, and safety [81]. In clinical trials (ClinicalTrials.gov ID: NCT02960893), troriluzole showed a reduction in the Scale for the Assessment and Rating of Ataxia (SARA) scores; however, it was also associated with increased serious and treatment-emergent adverse events [82]. A post hoc video-based machine learning analysis further suggested improved gait stability, particularly during tandem walking, although the findings were limited by incomplete data and potential methodological bias [81]. Despite these signals, regulatory approval was not granted due to insufficient and inconsistent evidence of clinical efficacy [83].

Despite these therapeutic advances, several challenges remain [72]. In addition, synthetic drug development is often constrained by adverse side effects, highlighting the need for safer therapeutic alternatives. Consequently, interest in natural medicines as complementary or alternative therapeutic agents for neurodegenerative diseases has increased [18,84]. Natural medicines remain a key source in drug discovery and have contributed substantially to modern therapeutics [19]. Preliminary findings also indicate that active compounds exhibit favorable binding affinities, supporting their potential as lead candidates for tuberculosis drug discovery [16]. Moreover, natural medicines targeting oxidative stress and protein aggregation may represent promising therapeutic strategies for SCA3 [19].

## 6. Complementary and Alternative Therapies for SCA3

Traditional medicines have long been used as complementary therapeutic agents in the management of various neurodegenerative diseases [84,85,86,87,88,89,90,91,92,93,94,95]. As no curative treatment currently exists for SCA3, and available pharmacological therapies provide only symptomatic relief and are limited by modest efficacy and adverse effects, there is an urgent need to explore alternative therapeutic strategies to slow disease progression [19]. In this context, natural medicines have been garnering significant attention as a potential treatment option because they are generally well tolerated, possess diverse active compounds, and can simultaneously modulate multiple pathogenic mechanisms involved in neurodegeneration, providing safer alternatives to synthetic medications [18,40]. Their widespread acceptance is reflected by the recognition of traditional medicine within the healthcare systems of 170 Member States, representing 88% of the World Health Organization (WHO) Member States, while approximately 80% of the global population has relied on herbal medicines to complement their primary healthcare needs over the past decade [96]. Moreover, natural medicines have long served as a valuable source of active compounds for drug discovery.

**Figure 3:**
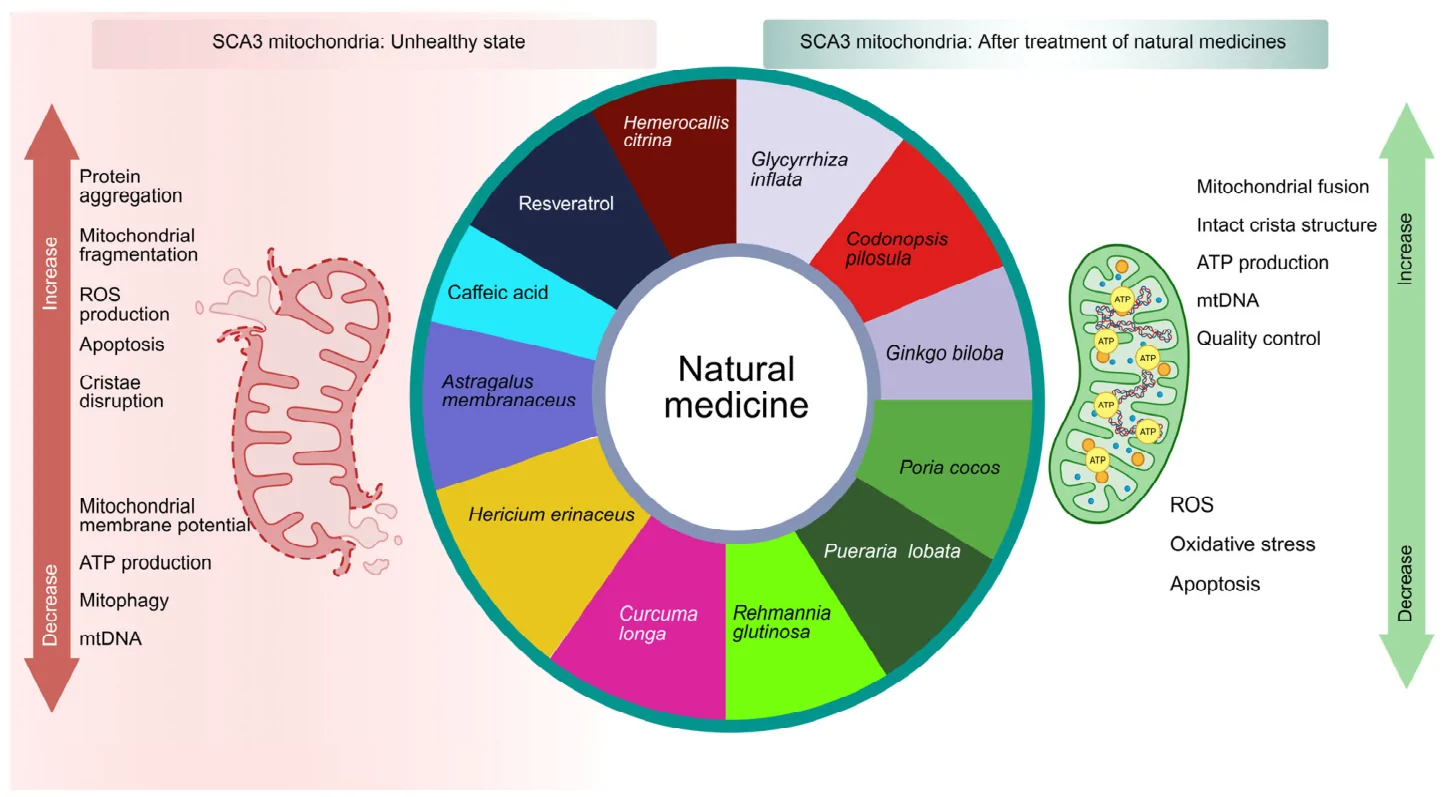
Modulation of mitochondrial dysfunction in SCA3 by natural medicines. ATP, adenosine triphosphate; mtDNA, mitochondrial DNA; ROS, reactive oxygen species; SCA3, spinocerebellar ataxia type 3. The schematic diagram was created using BioRender.com.

Given the central role of mitochondrial dysfunction in SCA3, secondary metabolites such as polyphenols, flavonoids, and carotenoids have emerged as promising therapeutic candidates due to their antioxidant [9,97] and mitochondria-protective properties, which reduce oxidative stress, preserve mitochondrial integrity, and improve cellular energy metabolism [98]. Figure 3 highlights natural medicines and their effects on mitochondrial dysfunction. In addition, mitochondria-targeting natural medicines have been shown to stimulate mitochondrial biogenesis, regulate mitochondrial fusion and fission, promote mitophagy, improve mitochondrial respiration and ATP production, and maintain mitochondrial homeostasis [99]. These multimodal effects suggest that natural medicines may mitigate the mitochondrial abnormalities underlying SCA3 pathogenesis.

Evidence from other ataxia models further supports the therapeutic potential of natural medicines in restoring mitochondrial function. In a 3-acetylpyridine-induced cerebellar ataxia model, treatment with *Hericium erinaceus* (Bull.: Fr.) Pers. extract improved mitochondrial function, as demonstrated by increased expression of cyt c oxidase subunit IV (COX-IV), a key component of the mitochondrial respiratory chain [100]. Furthermore, in fibroblasts derived from patients with Friedreich’s ataxia, *H. erinaceus* restored the reduced glutathione (GSH)/oxidized glutathione (GSSG) ratio following buthionine sulfoximine-induced oxidative stress, indicating improved mitochondrial redox homeostasis [101]. Collectively, these findings indicate that natural medicines can restore mitochondrial function through multiple complementary mechanisms and highlight their potential as promising therapeutic candidates for SCA3.

Mitochondrial dysfunction is implicated in the pathogenesis and progression of a broad range of diseases, including neurodegeneration, cardiovascular diseases, metabolic syndrome, and cancer [102]. As mitochondria regulate energy production, biosynthesis, calcium signaling, ROS, immune response, and apoptosis, alteration of mitochondrial activity can produce beneficial or harmful effects depending on the disease context and the metabolic state of the cells [103,104,105]. Although the therapeutic roles of natural medicines have been acknowledged to a certain extent in the management of SCA3 [9,17,18,19,20], the understanding of in-depth and fundamental mechanisms targeting mitochondrial dysfunction is still vague. Therefore, the narrative review aims to critically elucidate the current understanding of natural medicines targeting mitochondrial dysfunction in SCA3. The therapeutic consequences of mitochondrial modulation are specific to the cells and tissues impacted in SCA3.

A comprehensive literature search was conducted to identify preclinical studies investigating natural medicine-based interventions targeting mitochondrial dysfunction in SCA3. Electronic databases, namely the PubMed, Scopus, and Web of Science were systematically searched for relevant articles published between 1 January 2014 and 31 December 2025. The search strategy incorporated combinations of keywords: “spinocerebellar ataxia type 3 (SCA3),” OR “Machado-Joseph disease,” AND “mitochondrial morphology,” OR “mitochondrial function,” OR “mitochondrial homeostasis,” AND “natural medicine,” OR “natural product,” OR “herb,” OR “remedy,” OR “plant,” OR “compound,” OR “phytochemical,”. Peer-reviewed and full-length original research articles published in English were considered for inclusion. Studies unrelated to natural medicine-based interventions, studies that did not evaluate mitochondrial outcomes, conference abstracts, editorials, and articles containing insufficient or irrelevant data were excluded. Following the screening process, a total of seven articles encompassing twelve natural medicines fulfilled the inclusion criteria and were included in this review.

The natural medicines and their active compounds evaluated in preclinical models are summarized in Table 1. Although the changes in the level of molecular markers may not necessarily represent absolute recovery of mitochondrial function, the findings and mechanisms of action of reviewed studies could provide relevant knowledge to a certain extent in which natural medicines are involved in restoring mitochondrial function in SCA3. Indeed, restoration of proper mitochondrial function require simultaneous coordination of mitochondrial quality control for preserving mitochondrial health and ensuring cellular function. These include mitochondrial proteostasis that ensures proper protein folding and degradation, mitochondrial biogenesis that guards the synthesis of new mitochondria, mitochondrial dynamics that encompass fusion and fission processes, and mitophagy, the selective autophagic removal of damaged mitochondria.

Importantly, the strength of evidence for mitochondrial modulation depends on the endpoints measured. Measurement of direct mitochondrial effects provides solid evidence of involvement of mitochondrial function, membrane potential, ATP production, mitochondrial dynamics, mitophagy, and mitochondrial antioxidant defense. In contrast, measurement of indirect mitochondrial effects provides information on changes in the upstream regulators or systemic antioxidant responses, and level of oxidative stress without any evidence of mitochondrial association.

**Figure 4:**
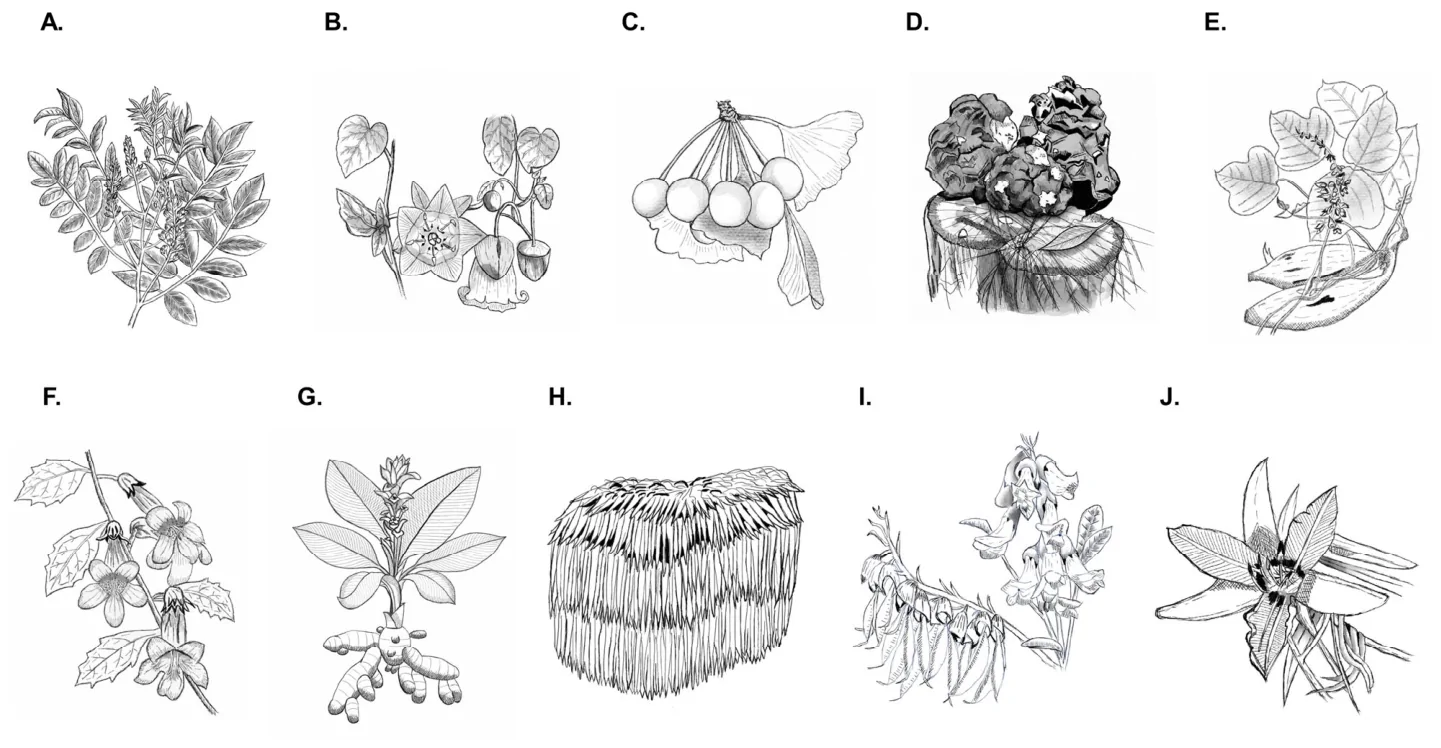
Natural medicines for SCA3. (**A**) *Glycyrrhiza inflata* Batalin; (**B**) *Codonopsis pilosula* (Franch.) Nannf.; (**C**) *Ginkgo biloba* L.; (**D**) *Poria cocos* (Schw.) Wolf; (**E**) *Pueraria lobata* (Willd.) Ohwi; (**F**) *Rehmannia glutinosa* Libosch.; (**G**) *Curcuma longa* L.; (**H**) *Hericium erinaceus* (Bull.: Fr.); (**I**) *Astragalus membranaceus* (Fisch.) Bunge; (**J**) *Hemerocallis citrina* Baroni.

**Figure 5:**
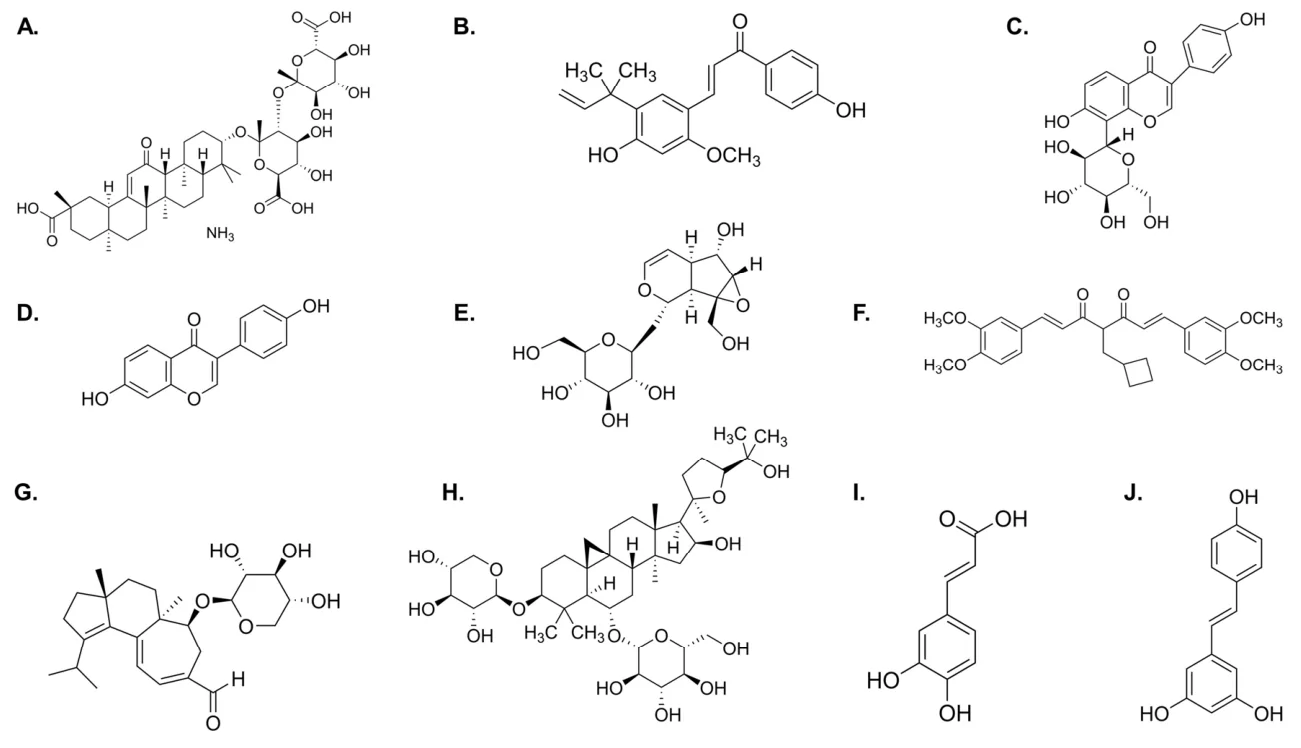
Active compounds for SCA3. (**A**) Ammonium glycyrrhizinate (AMGZ); (**B**) Licochalcone A; (**C**) Puerarin; (**D**) Daidzein; (**E**) Catalpol; (**F**) JM17 (a curcumin analog); (**G**) Erinacine A; (**H**) Astragaloside IV; (**I**) Caffeic acid; (**J**) Resveratrol.

The external morphological features of the natural medicines are presented in Figure 4; namely *Glycyrrhiza inflata* Batalin (Figure 4A), *Codonopsis pilosula* (Franch.) Nannf. (Figure 4B), *Ginkgo biloba* L. (Figure 4C), *Poria cocos* (Schw.) Wolf (Figure 4D), *Pueraria lobata* (Willd.) Ohwi (Figure 4E), *Rehmannia glutinosa* Libosch. (Figure 4F), *Curcuma longa* L. (Figure 4G), *H. erinaceus* (Figure 4H), *Astragalus membranaceus* (Fisch.) Bunge (Figure 4I), and *Hemerocallis citrina* Baroni (Figure 4J). The active compounds of the identified natural medicines are presented in Figure 5; namely the ammonium glycyrrhizinate (AMGZ) (Figure 5A), licochalcone A (Figure 5B), puerarin (Figure 5C), daidzein (Figure 5D), catalpol (Figure 5E), JM17 (a curcumin analog) (Figure 5F), erinacine A (Figure 5G), astragaloside IV (Figure 5H), caffeic acid (Figure 5I), and resveratrol (Figure 5J).

### 6.1. Glycyrrhiza Inflata

*Glycyrrhiza inflata* is a medicinal licorice species used in traditional medicine for centuries [113]. Also known as licorice, Chinese licorice, Manchurian licorice, or Asiatic licorice [114,115], the roots of *Glycyrrhiza* species have long been used to treat a variety of conditions, including peptic ulcers, hepatitis C, diabetes, and pulmonary and skin disorders [106].

Studies have shown that licorice contains major active compounds, primarily triterpenoids and flavonoids. Among them, licochalcone A is a characteristic flavonoid of *G. inflata* [113]. Both licochalcone A and glycyrrhizin have been widely reported to exhibit anti-inflammatory activity [116], antioxidant effects [117], and neuroprotective properties [118]. In addition, AMGZ, another important derivative, is well known for its anti-inflammatory effects [119]. Additionally, AMGZ demonstrated protective effects in vitro in SH-SY5Y cells exposed to high-glucose-induced stress. Specifically, it reduced cytotoxicity, prevented mitochondrial fragmentation, and preserved mitochondrial integrity and function [120]. These findings highlight its potential role in supporting mitochondrial protection and repair mechanisms under hyperglycemic conditions.

In human embryonic kidney (HEK)-293 cells expressing mutant ataxin-3 with 75 polyQ repeats, 1 µM AMGZ and 10 µM licochalcone A reduced mutant ataxin-3 aggregation and apoptosis, while improving mitochondrial-related functions and activating mitochondrial biogenesis and antioxidant activity [106]. Importantly, this study extended the previously recognised antioxidant and neuroprotective properties of these compounds by demonstrating their potential to counteract mutant ataxin-3-associated mitochondrial dysfunction and proteotoxicity in SCA3.

### 6.2. Curcuma Longa

*Curcuma longa*, commonly known as turmeric, is a medicinal herb in the ginger family cultivated in tropical regions of South and Southeast Asia [121]. It has been used for centuries in traditional Indian and Chinese medicine due to its anti-inflammatory [122], antioxidant [123], antidiabetic [124], and blood circulation-promoting properties [125], and has been applied in the treatment of various conditions, including skin diseases, infections, stress, and depression [121,125].

Turmeric contains numerous active compounds, including alkaloids, tannins, phenolic compounds, terpenoids, phytosterols, saponins, and flavonoids [122]. One such compound, JM17, is a bioavailable curcumin analog that promotes ubiquitin–proteasome-mediated degradation of the androgen receptor (AR) [108,126]. The JM17 has demonstrated therapeutic potential in polyQ neurodegenerative disorders by promoting the clearance of toxic polyQ protein aggregates and improving motor function, supporting its potential as a therapeutic candidate for SCA3 [126].

Wu et al. demonstrated that 0.3, 1, and 5 µm JM17 alleviates mitochondrial dysfunction, including reduced oxygen consumption, impaired ATP-linked respiration, and increased mitochondrial ROS production, in human neuroblastoma cells, SK-N-SH-MJD78 expressing mutant ataxin-3 with 78 polyQ repeats. Treatment with JM17 significantly restores mitochondrial respiration, enhances ATP production, and reduces oxidative stress, thereby improving mitochondrial function in SCA3 cellular models [108].

### 6.3. Hericium Erinaceus

*Hericium erinaceus* belongs to the *Hericium* genus, which includes several species such as *Hericium americanum* and *Hericium coralloides*. Among them, *H. erinaceus* is particularly notable due to its distinctive morphology and well-documented medicinal properties [86,100,101,127,128,129,130]. Commonly known as lion’s mane mushroom, monkey head, or “pom-pom mushroom” due to its unique appearance, *H. erinaceus* is an edible and medicinal fungus that has been widely used in traditional Chinese medicine for centuries [131]. It is naturally distributed across Europe, Asia, and North America [132]. *Hericium erinaceus* contains several major classes of active compounds, including polysaccharides, terpenoids, and phenolic compounds [131]. The polysaccharide fraction is primarily composed of β-glucans, which are associated with immunomodulatory and anti-inflammatory activities [132,133]. Terpenoids, particularly erinacines (cyathane-type diterpenoids), are key secondary metabolites linked to neuroprotective effects and the stimulation of nerve growth factor (NGF) synthesis [134]. Phenolic compounds are present in smaller quantities but contribute significantly to antioxidant activity by reducing oxidative stress and enhancing cellular redox balance [133,135]. The therapeutic potential of *H. erinaceus* has been widely demonstrated in preclinical studies. It has been shown to promote neuronal differentiation [136,137], peripheral nerve regeneration [138,139], enhance neurogenesis [129], and exert anti-inflammatory effects in neurodegenerative models [100,140,141]. In addition, its neuroprotective properties have been reported in various experimental systems [86,128], highlighting its ability to support neuronal survival and function under pathological conditions [142].

Further evidence comes from Wu et al. who investigated the effects of erinacine A-enriched mycelial extract in cellular and *Drosophila* models of SCA3 under oxidative stress. In SK-N-SH-MJD78 cells expressing mutant ataxin-3 with 78 polyQ repeats, 1.25, 2.5, and 5 µg/mL erinacine A-enriched mycelial extract reduced tert-butyl hydroperoxide-induced apoptosis and mutant ataxin-3 protein aggregation, preserved mitochondrial membrane potential, and modulated apoptosis-related proteins, including Bax, B-cell lymphoma 2 (Bcl-2), caspases, and poly (ADP-ribose) polymerase (PARP). In the ELAV-SCA3tr-Q78 *Drosophila* model expressing mutant ataxin-3 with 78 polyQ repeats, 0.5% and 1% *w*/*w* erinacine A-enriched mycelial extract significantly improved survival and locomotor performance, while attenuating oxidative stress, protein aggregation, and mitochondria-associated apoptotic signaling. Collectively, these findings suggest that erinacine A-enriched mycelial extract exerts protective effects against oxidative stress and mitochondrial dysfunction-associated neurodegeneration in SCA3 models [109].

### 6.4. Astragalus Membranaceus

*Astragalus membranaceus*, commonly known as Huangqi, is a traditional medicinal herb whose dried root (Astragali radix) contains a diverse range of active constituents, including flavonoids, polysaccharides, and triterpenoid saponins [143,144], with astragaloside IV identified as a key active compound [145]. Astragaloside IV has attracted considerable attention due to its favorable safety profile demonstrated in preclinical and clinical studies [145,146], as well as its broad pharmacological activities, including neuroprotective effects [147], antioxidant [110], immunoregulatory [148], neuroregenerative potential [149], and anti-inflammatory effects [150], making it a promising therapeutic candidate for neurodegenerative diseases.

Furthermore, in vitro evidence from SK-N-SH-MJD78 cells expressing mutant ataxin-3 with 78 polyQ repeats, demonstrates that 12.5, 25, and 50 µM astragaloside IV attenuate mitochondrial dysfunction by reducing oxidative stress, enhancing antioxidant defense, and improving mitochondrial bioenergetics, as reflected by increased GSH, superoxide dismutase (SOD), glutathione peroxidase (GPx), and catalase (CAT) levels, as well as improved mitochondrial respiration and membrane potential. These effects were accompanied by reduced excessive mitochondrial fission and enhanced clearance of mutant ataxin-3, suggesting coordinated modulation of mitochondrial function, oxidative stress, and proteostasis in the SCA3 cellular model [110].

### 6.5. Hemerocallis Citrina

*Hemerocallis citrina*, a member of the Liliaceae family, is a traditional Chinese medicine commonly known as “Forgetting Sadness Grass” [151]. The *H. citrina* is widely consumed as a food, particularly in China, Japan, and Korea. Its flowers are used in various traditional dishes, including hot and sour soup, daylily soup, Buddha’s delight, and moo shu pork. In addition to its culinary uses, *H. citrina* has a long history of medicinal application [152]. It is well for its antidepressant [153], neuroprotective [151], anti-inflammatory [154], and anti-aging [155]. Furthermore, Fernandes et al. demonstrated that 0.750 and 1.000 mg/mL of 50% ethanol extract and 0.250, 0.500, 0.750, and 1.000 mg/mL of 30% ethanol extract improved locomotor deficits in transgenic *C. elegans* expressing mutant human ataxin-3 with 130 polyQ repeats, with 0.250 mg/ml of 30% ethanol extract showing the most potent activity. However, the extracts failed to enhance mitochondrial unfolded protein response (UPRmt) or significantly reduce mutant ataxin-3 aggregation in neurons [111]. Indeed, UPRmt has been described as a compensatory response that reduces mitochondrial damage in aging and neurodegenerative disease. However, at some point, once the degree of mitochondrial dysfunction reaches a critical level, endogenous activation of the UPRmt is insufficient for counteracting the insults.

### 6.6. Other Natural Medicines

In a study by Chen et al., 10 μg/mL crude aqueous extracts of *C. pilosula*, *G. biloba*, *P. cocos*, *P. lobata*, and *R. glutinosa* had been found to reduce mutant ataxin-3 aggregation in the green fluorescent protein (GFP)-HEK-293 cells expressing mutant ataxin-3 with 75 CAG repeats. Subsequently, the extracts were evaluated in differentiated human SH-SY5Y cells expressing mutant ataxin-3 with 75 CAG repeats. The treatment increased neurite length, process number, and branch points while reducing apoptosis-related markers, suggesting a potential neuroprotective effect [107].

In addition, extracts of *P. lobata* and *R. glutinosa* reduced intracellular ROS and restored mitochondrial antioxidant defense in GFP-HEK-293 cells expressing mutant ataxin-3 with 75 CAG repeats. Remarkably, 0.1 μM puerarin and daidzein, the active compounds of *P. lobata* and catalpol (0.1 μM) isolated from *R. glutinosa* also showed similar activities. Meanwhile, 0.1 μM puerarin and daidzein, and 100 nM catalpol also increased neurite outgrowth and reduced mutant ataxin-3 aggregation and apoptosis-related signaling in the differentiated GFP-SH-SY5Y cells expressing mutant ataxin-3 with 75 CAG repeats [107].

### 6.7. Other Active Compounds

Individual active compounds have also been investigated for their potential neuroprotective effects in SCA3, particularly through modulation of mitochondrial function and oxidative stress pathways. These compounds also exhibit notable antioxidant and neuroprotective properties.

Caffeic acid is a naturally occurring hydroxycinnamic acid and polyphenolic compound widely distributed in vegetables, fruits, and medicinal herbs [156]. It is biosynthesized in plants via the phenylpropanoid pathway through hydroxylation of p-coumaric acid [157]. Caffeic acid has attracted considerable attention due to its broad pharmacological activities, including antioxidant [158], antidiabetic [159], and neuroprotective effects [157]. In addition, caffeic acid preserves mitochondrial function by reducing mitochondrial oxidative stress, maintaining mitochondrial integrity, and preventing mitochondrial dysfunction [160]. Resveratrol is a polyphenolic phytoalexin found in at least 72 plant species, including red wine, mulberries, peanuts, and rhubarb [161,162]. It is reported to possess multiple pharmacological activities, including cardioprotective [163], anti-inflammatory [164], and anti-aging effects [165]. Following absorption via passive diffusion and carrier-mediated transport, resveratrol is rapidly metabolized into glucuronide and sulfate conjugates [166]. Resveratrol has been shown to enhance mitochondrial biogenesis and activate AMP-activated protein kinase (AMPK) and PPARGC1A signaling, leading to improved mitochondrial function and energy metabolism [167]. In the SCA3 model, both caffeic acid and resveratrol demonstrate mitochondrial protective effects. Furthermore, in vitro studies using SK-N-SH-MJD78 cells expressing mutant ataxin-3 with 78 polyQ repeats treated with 3 μM of either compound showed reduced ROS production and restoration of mitochondrial membrane potential, indicating improved mitochondrial function [112].

## 7. Mechanisms of Action of Natural Medicines Targeting Mitochondrial Dysfunction in SCA3

### 7.1. Attenuation of Mitochondrial Oxidative Stress

**Figure 6:**
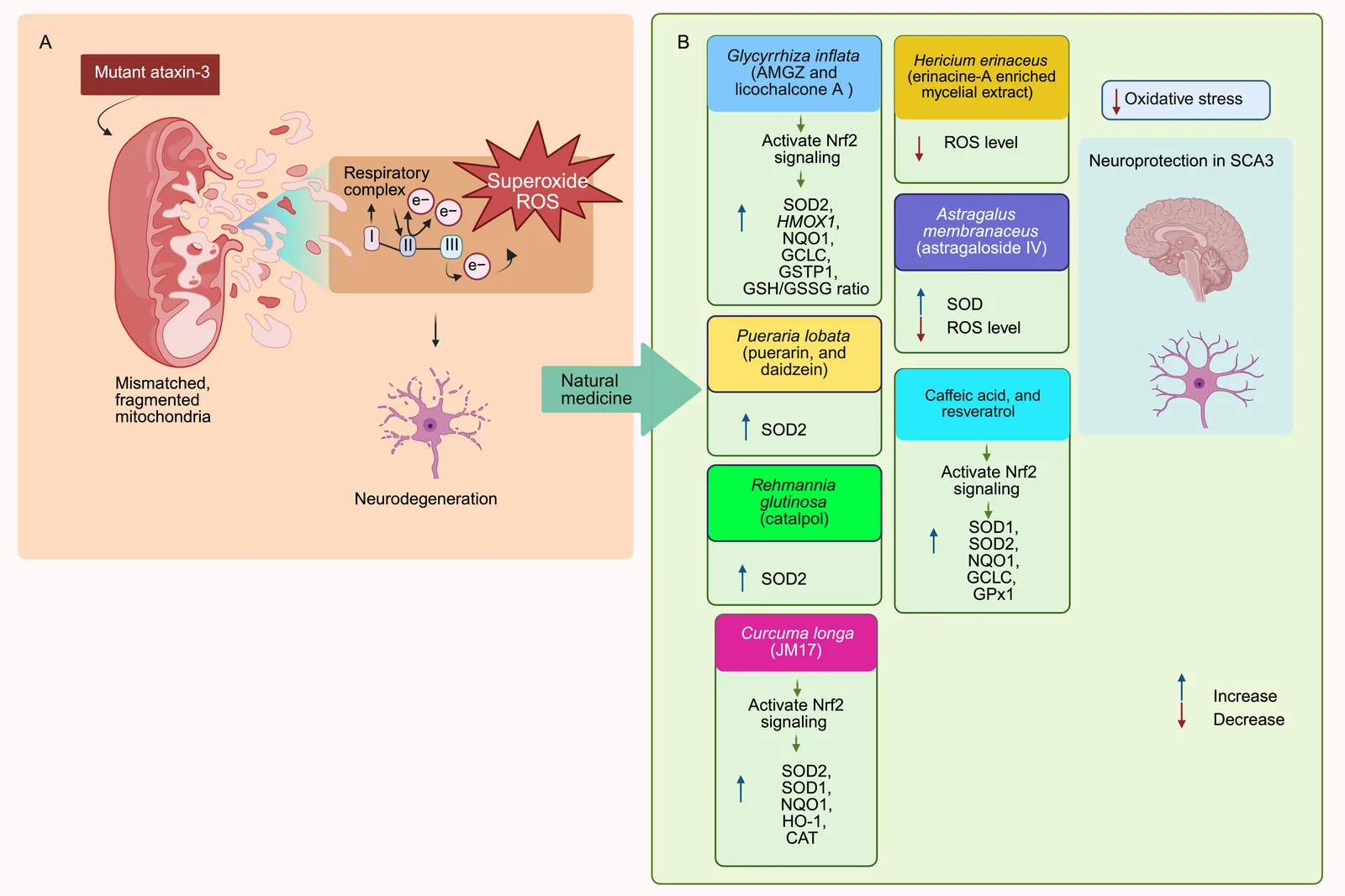
Natural medicines targeting mitochondrial oxidative stress in SCA3 to promote neuroprotection. (**A**) Dysfunctional, fragmented mitochondria release superoxide and ROS via respiratory complexes I-III, driving neurodegeneration. (**B**) Natural medicines counteract this pathogenesis through distinct mechanisms to support neuroprotection: (i) AMGZ and licochalcone A activate Nrf2 signaling to upregulate antioxidant proteins (SOD2, *HMOX1*, NQO1, GCLC, GSTP1, GSH/GSSG); (ii) Puerarin, daidzein, and catalpol increases SOD2; (iii) JM17 activates Nrf2 signaling to upregulate antioxidant protein (SOD1, SOD2, NQO1, HO-1, CAT); (iv) Erinacine A-enriched mycelial extract reduces ROS level; (v) Astragaloside IV increases SOD and reduce ROS level; and (vi) Caffeic acid and resveratrol activate Nrf2 signaling to upregulate antioxidant protein (SOD1, SOD2, NQO1, GCLC, GPx1). AMGZ, ammonium glycyrrhizinate; CAT, catalase; e-, electron; GCLC, glutamate-cysteine ligase catalytic subunit; GPx1, glutathione peroxidase 1; GSH, reduced glutathione; GSSG, oxidized glutathione; GSTP1, glutathione S-transferase pi 1; *HMOX1* (heme oxygenase 1); HO-1, heme oxygenase 1; NQO1, NAD(P)H quinone dehydrogenase 1; Nrf2, nuclear factor erythroid 2-related factor 2; ROS, reactive oxygen species; SOD, superoxide dismutase; SOD1, superoxide dismutase 1; SOD2, superoxide dismutase 2. The schematic diagram was created using BioRender.com.

Oxidative stress occurs when ROS production exceeds the capacity of intracellular antioxidant defense systems [18]. In neurons, mitochondria are the primary source of ROS, mainly generated through electron leakage from respiratory chain complexes I, II, and III during oxidative phosphorylation, leading to superoxide (O_2_•^−^) formation [168,169] (Figure 6A).

Active compounds have been widely reported to restore mitochondrial redox homeostasis by targeting antioxidant signaling pathways [170]. In SCA3 models, AMGZ and licochalcone A activate the NF-E2-related factor 2 (Nrf2)–antioxidant responsive element (ARE) pathway, leading to the upregulation of downstream antioxidant gene and proteins, including heme oxygenase 1 (*HMOX1* and HO-1), NAD(P)H quinone dehydrogenase 1 (NQO1), glutamate-cysteine ligase catalytic subunit (GCLC), glutathione S-transferase pi 1 (GSTP1), and the mitochondrial antioxidant enzyme, superoxide dismutase 2 (SOD2). This response was associated with reduced ROS levels and improved GSH/GSSG redox status. Importantly, Chen et al. further linked this antioxidant response to mitochondrial biogenesis and mutant ataxin-3 proteostasis by showing that AMGZ and licochalcone A increased peroxisome proliferator-activated receptor gamma coactivator 1-alpha (PPARGC1A) expression and activity, while PPARGC1A knockdown attenuated their anti-aggregation effects. These findings suggest that coordinated activation of Nrf2–ARE and PPARGC1A pathways may connect restoration of redox homeostasis with suppression of mutant ataxin-3 aggregation, providing a mechanistic link between mitochondrial dysfunction and proteotoxicity in SCA3 [106].

Moreover, crude aqueous extracts of *P. lobata* and *R. glutinosa*, together with their active compounds puerarin, daidzein, and catalpol, reduced ROS levels and increased SOD2 protein expression, indicating enhanced mitochondrial antioxidant defence [107]. Similarly, JM17 attenuates mitochondrial oxidative stress in SCA3 cellular models by reducing mitochondrial ROS, increasing SOD2, superoxide dismutase 1 (SOD1), CAT, heme oxygenase 1 (HO-1), and NQO1 expression, and elevating glutathione levels through activation of the Nrf2–ARE pathway [108]. Notably, the protective effects of JM17 were not proportional to Nrf2 activation, suggesting that additional mechanisms may contribute to its mitochondrial effects. The detection of JM17 in mitochondria further raises the possibility of direct mitochondrial actions. However, whether JM17 modulates Drp1/mitochondrial fission 1 protein (FIS1)-mediated fission, MFN1/2- and OPA1-mediated fusion, or PINK1/Parkin-dependent mitophagy, and how these processes interact with its established autophagy-mediated clearance of mutant ataxin-3, remains unclear.

In addition, erinacine A-enriched mycelial extract reduces mitochondrial ROS levels in SCA3-related neuronal models, supporting mitochondrial redox protection [109]. Astragaloside IV enhances antioxidant capacity by increasing SOD activity. Thereby attenuating mitochondrial oxidative stress [110]. Caffeic acid and resveratrol increased Nrf2 signaling activation and antioxidant defense in SK-N-SH-MJD78 cells, including SOD1, SOD2, NQO1, GCLC and GPx1, thereby supporting cellular and mitochondrial antioxidant protection. Notably, Nrf2 silencing attenuated these effects and impaired autophagy-mediated clearance of mutant ataxin-3, suggesting that Nrf2 signaling links antioxidant defense with mitochondrial protection and proteostasis in SCA3 [112].

Collectively, attenuation of mitochondrial oxidative stress represents a central therapeutic mechanism of active compounds in SCA3, mainly through suppression of mitochondrial ROS generation and reinforcement of antioxidant defense systems, particularly via Nrf2-ARE-mediated signaling and mitochondrial SOD2 (Figure 6B).

### 7.2. Enhancement of Mitochondrial Biogenesis and Bioenergetics

**Figure 7:**
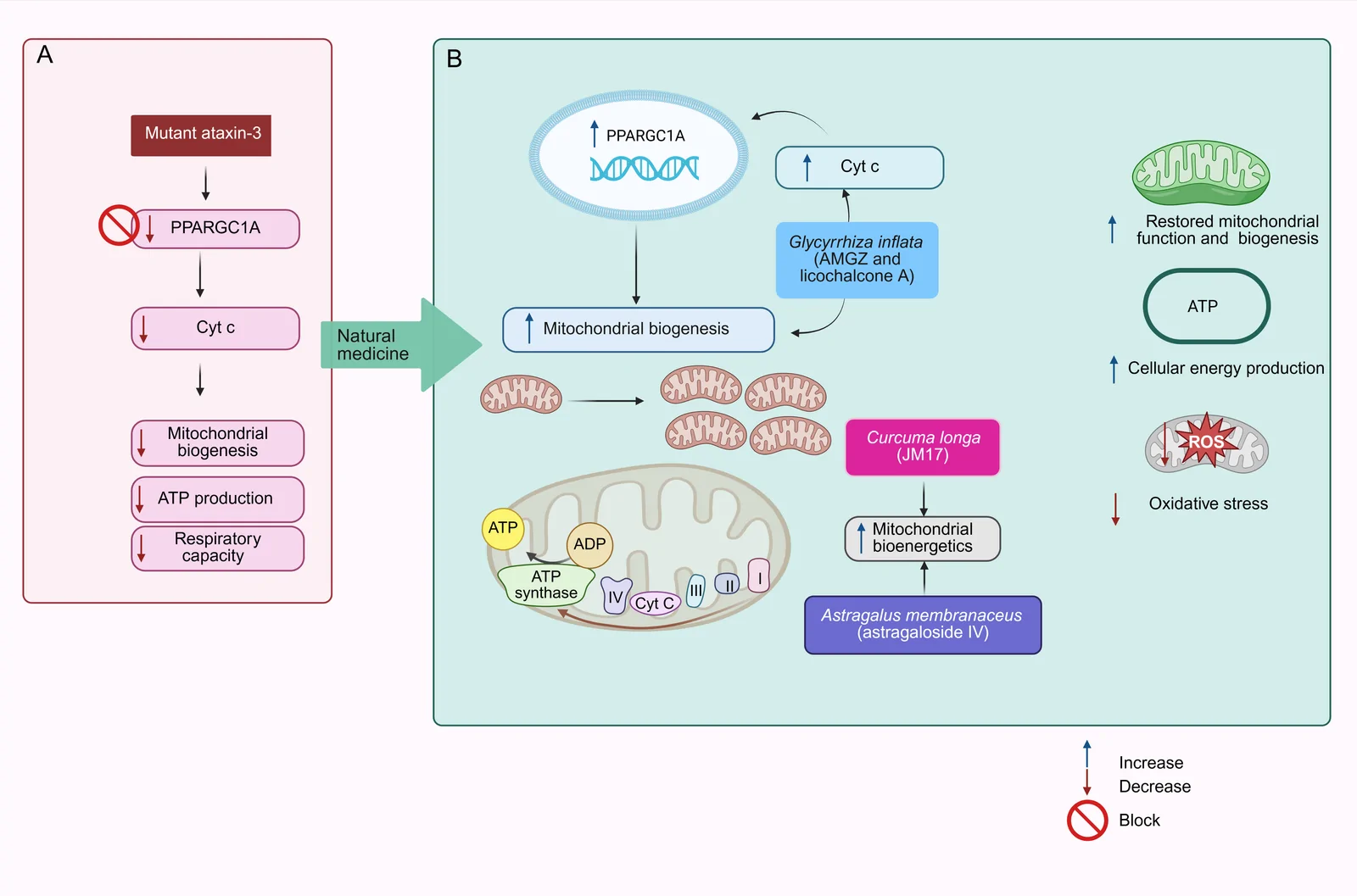
Natural medicines targeting mitochondrial biogenesis and bioenergetics in SCA3. (**A**) Mutant ataxin-3 suppresses PPARGC1A, leading to reduced cyt c expression, impaired mitochondrial biogenesis, decreased ATP production, and diminished respiratory capacity. (**B**) Natural medicines reverse these deficits through distinct mechanisms: (i) AMGZ and licochalcone A increase PPARGC1A levels to promote mitochondrial biogenesis; (ii) JM17 enhances mitochondrial respiration; and (iii) astragaloside IV increases OCR and OXPHOS. Ultimately, these compounds restore mitochondrial function, increase cellular energy output, and reduce oxidative stress. ADP, adenosine diphosphate; ATP, adenosine triphosphate; AMGZ, ammonium glycyrrhizinate; OCR, oxygen consumption rate; OXPHOS, oxidative phosphorylation; PPARGC1A, peroxisome proliferator-activated receptor gamma coactivator 1-alpha; ROS, reactive oxygen species. The schematic diagram was created using BioRender.com.

The PPARGC1A is a key regulator of mitochondrial biogenesis and antioxidant response genes [171]. Loss of PPARGC1A has been associated with neurodegeneration and mitochondrial dysfunction, as demonstrated in PPARGC1A-null mice, which develop spongiform neurodegeneration in selective brain regions [106,172] (Figure 7A). Moreover, reduced PPARGC1A signaling has been implicated in clinically distinct neurodegenerative diseases [173].

In the ATXN3/Q75 SCA3 model, mutant ataxin-3 expression suppresses mitochondrial biogenesis-related signaling, as evidenced by decreased PPARGC1A expression and downregulation of downstream targets such as cyt c. Treatment with AMGZ and licochalcone A restores PPARGC1A expression, indicating reactivation of mitochondrial biogenesis [106]. Similarly, JM17 improves mitochondrial bioenergetics by enhancing mitochondrial respiration in SCA3 cellular models, including increased basal respiration and ATP-linked respiration [108]. This restoration of mitochondrial respiratory capacity suggests improved cellular energy production and mitochondrial functional efficiency.

In addition, astragaloside IV enhances mitochondrial bioenergetics by increasing oxygen consumption rates (OCR) and improving oxidative phosphorylation (OXPHOS) capacity. Specifically, astragaloside IV upregulates maximal mitochondrial respiratory capacity, thereby improving overall bioenergetic performance and supporting cellular energy homeostasis [110] (Figure 7B).

### 7.3. Preservation of Mitochondrial Membrane Potential and Mitochondrial Integrity

**Figure 8:**
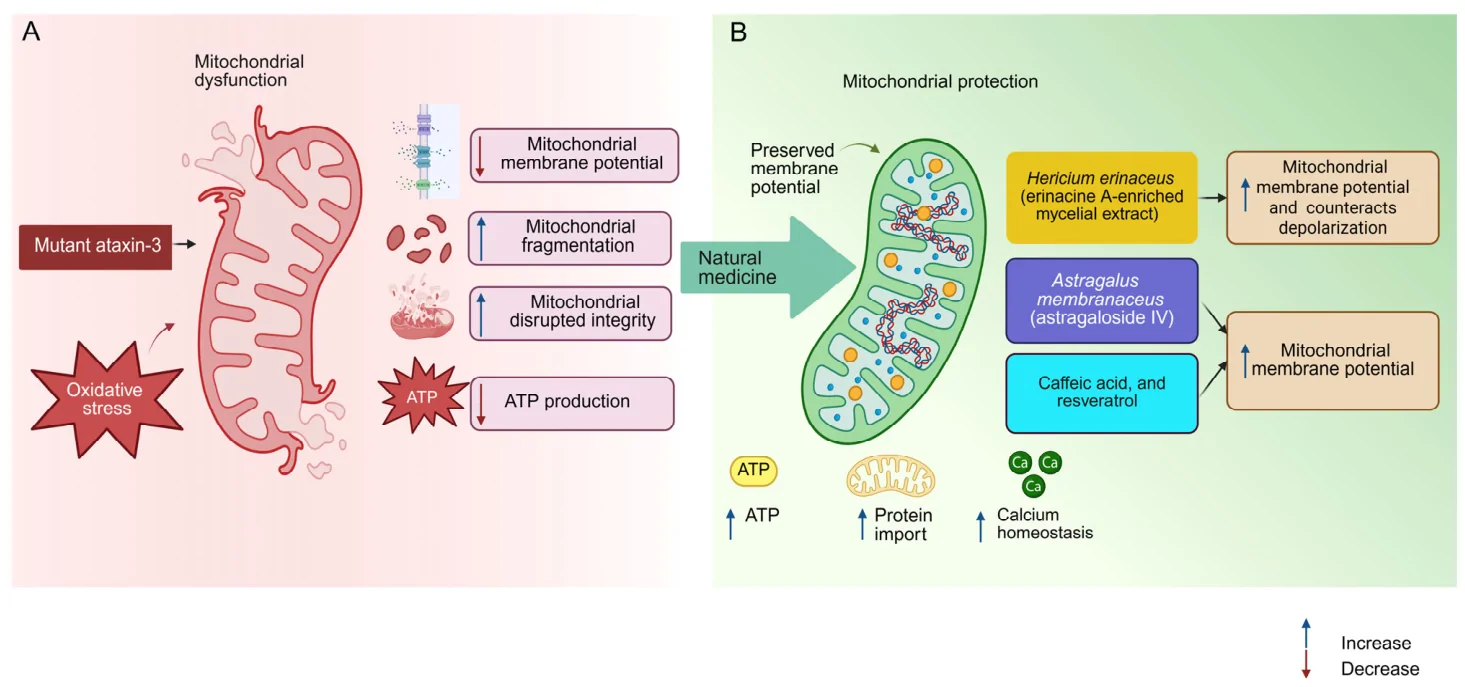
Natural medicines targeting mitochondrial membrane potential damage in SCA3. (**A**) Mutant ataxin-3 impairs mitochondrial membrane potential, mitochondrial structure, and ATP output. (**B**) Intervention with natural medicines (erinacine A-enriched mycelial extract, astragaloside IV, caffeic acid, and resveratrol) prevents mitochondrial depolarization and fragmentation, thereby preserving membrane potential, increasing ATP synthesis, promoting protein import, and maintaining calcium homeostasis. ATP, adenosine triphosphate. The schematic diagram was created using BioRender.com.

Maintenance of mitochondrial membrane potential is essential for ATP production, mitochondrial protein import, calcium homeostasis, and overall mitochondrial integrity [61]. Loss of mitochondrial membrane potential is a key indicator of mitochondrial dysfunction and is closely associated with oxidative stress-induced mitochondrial damage [174] (Figure 8A).

Erinacine A-enriched mycelial extract has been reported to restore mitochondrial membrane potential, thereby counteracting oxidative stress-induced mitochondrial depolarization. This finding suggests that erinacine A-enriched mycelial extract protects mitochondria against oxidative damage and helps preserve mitochondrial integrity and function [109]. Similarly, astragaloside IV has been shown to preserve mitochondrial membrane potential and maintain mitochondrial structural integrity in SCA3 cellular models. Treatment with astragaloside IV restored mitochondrial membrane potential, indicating protection against mitochondrial depolarization [110]. Furthermore, in SK-N-SH-MJD78 cells, mutant ataxin-3 induced a decrease in mitochondrial membrane potential. However, treatment with caffeic acid or resveratrol restored mitochondrial membrane potential, indicating recovery of mitochondrial function [112] (Figure 8B).

### 7.4. Regulation of Mitochondrial Dynamics

**Figure 9:**
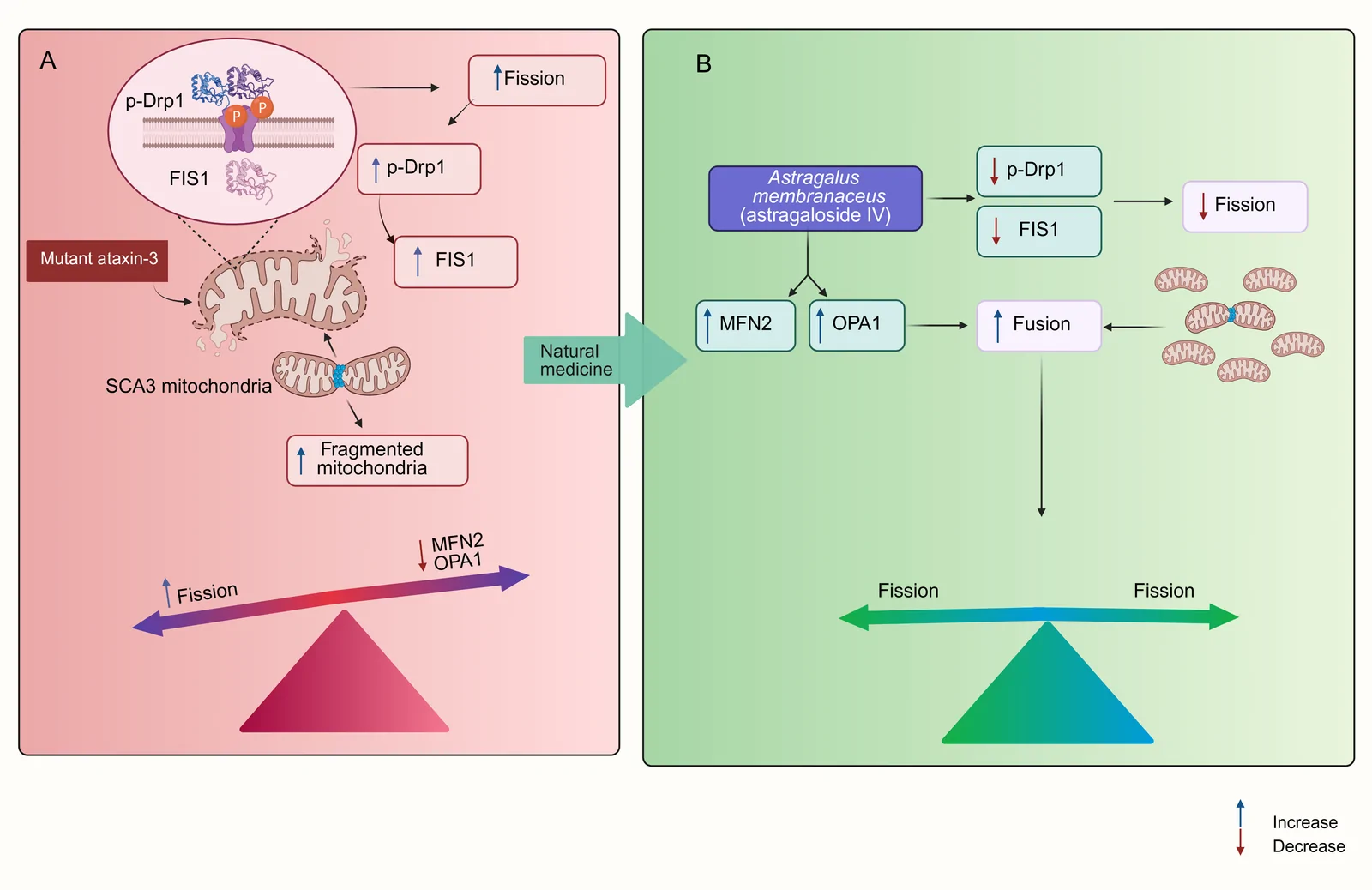
Natural medicines targeting mitochondrial dynamics imbalance in SCA3. (**A**) In SCA3 mitochondria, upregulated fission proteins (p-Drp1 and FIS1) and downregulated fusion markers (MFN2 and OPA1) shift the balance toward excessive mitochondrial fission and fragmentation. (**B**) Intervention with natural medicines (astragaloside IV) restores mitochondrial dynamics balance by suppressing p-Drp1 and FIS1 to attenuate fission while promoting MFN2 and OPA1 expression to enhance fusion. FIS1, mitochondrial fission 1 protein; MFN2, mitofusin 2; OPA1, optic atrophy 1; P, phosphorylation; p-Drp1, phosphorylated dynamin-related protein 1; SCA3; spinocerebellar ataxia type 3. The schematic diagram was created using BioRender.com.

The SK-N-SH-MJD78 cells show increased mitochondrial fragmentation associated with elevated phosphorylated dynamin-related protein 1 (p-Drp1) and mitochondrial FIS1 protein (Figure 9A). However, astragaloside IV regulates mitochondrial dynamics in the SCA3 model by suppressing excessive mitochondrial fission and restoring fusion balance. Astragaloside IV treatment downregulates p-Drp1 and FIS1, reducing abnormal mitochondrial fission. In contrast, mitochondrial fusion proteins such as MFN2 and optic atrophy 1 (OPA1) show a slight shift toward increased fusion capacity. Overall, Lin et al. showed astragaloside IV shifts mitochondrial dynamics toward a more fused and stable mitochondrial network, contributing to improved mitochondrial integrity in SCA3 cells. [110] (Figure 9B). However, whether enhanced antioxidant defense contributes to these mitochondrial benefits through mitophagy-related pathways (PINK1/Parkin, BNIP3, BCL2 interacting protein 3 like (BNIP3L), and FUN14 domain-containing protein 1 (FUNDC1)) and mitochondrial biogenesis (PPPARGC1A, nuclear respiratory factor 1 (NRF1), and mitochondrial transcription factor A (TFAM)) remains unclear.

### 7.5. Suppression of Mitochondria-Mediated Apoptosis

**Figure 10:**
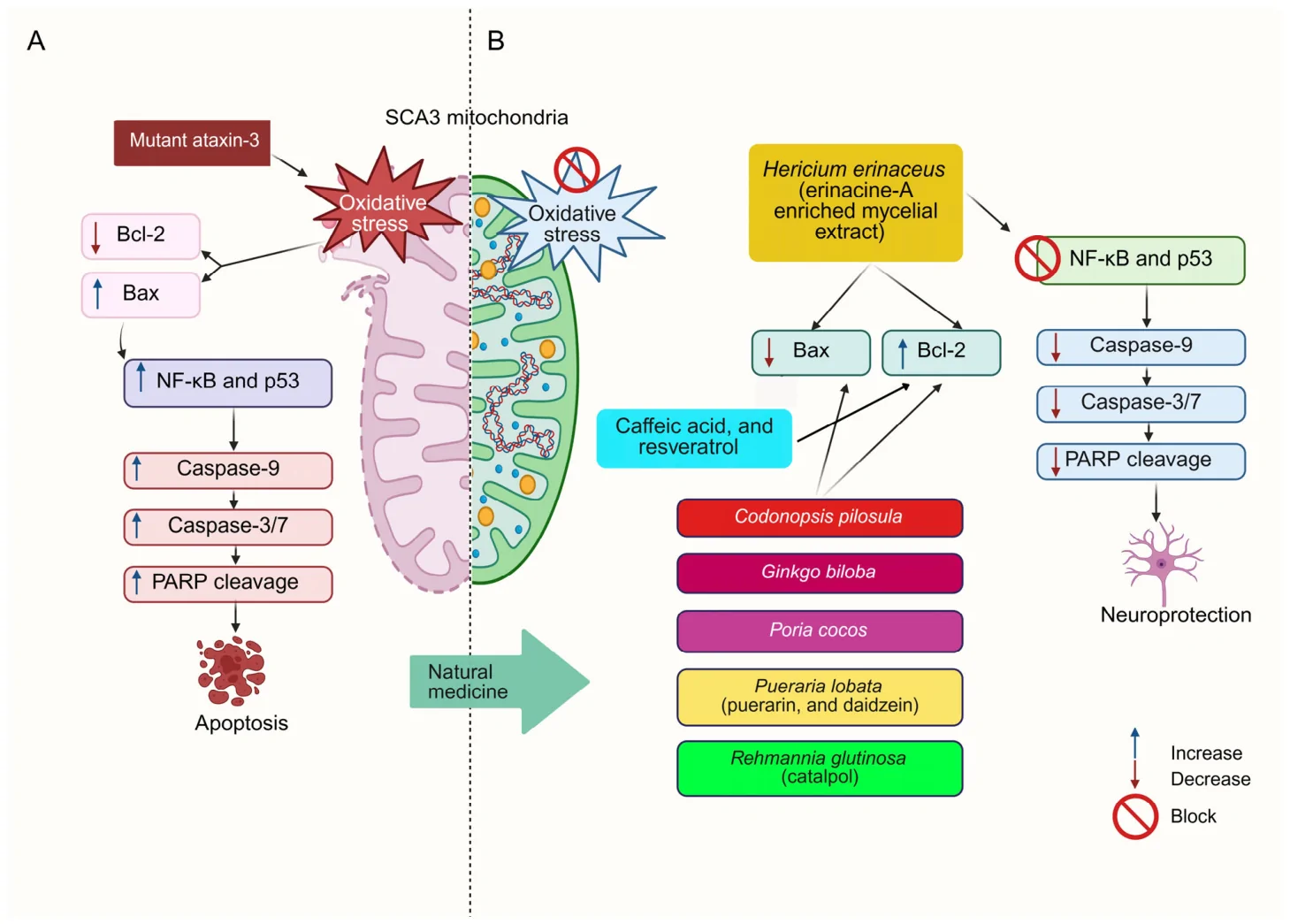
Natural medicines targeting apoptosis in SCA3. (**A**) Mutant ataxin-3 and oxidative stress downregulate Bcl-2, upregulate Bax, and trigger cyt c release, activating the caspase-9/3/7, and PARP cleavage cascade to induce apoptosis. (**B**) Natural medicines counteract this pathogenesis through distinct mechanisms: (i) Caffeic acid and resveratrol modulate Bcl-2 expression, contributing to suppression of the apoptotic cascade and neuronal protection; (ii) Erinacine A-enriched mycelial extract mitigates oxidative stress, restores the Bax/Bcl-2 balance, blocks NF-κB and p53, and inhibits activation of the downstream caspase cascade and PARP cleavage, thereby promoting neuronal survival; and (iii) *C. pilosula, G. biloba, P. cocos,* puerarin, daidzein, and catalpol restore the Bax/Bcl-2 balance, contributing to suppression of the apoptotic cascade and neuronal protection. Bcl-2, B-cell lymphoma 2; Bax, Bcl-2-associated X protein; cyt c, cytochrome c; NF-κB, nuclear factor-κB; PARP, poly (ADP-ribose) polymerase; SCA3, spinocerebellar ataxia type 3. The schematic diagram was created using BioRender.com.

Mutant ataxin-3 accumulation promotes oxidative stress and mitochondrial dysfunction, which can contribute to mitochondria-mediated apoptotic signaling in SCA3 models. Chen et al. reported that crude aqueous extracts of *C. pilosula*, *G. biloba*, *P. cocos*, *P. lobata*, and *R. glutinosa*, as well as the active compounds puerarin, daidzein, and catalpol, modulated apoptosis-related markers, including increased Bcl-2 and decreased Bax and caspase-3 expression, suggesting attenuation of apoptotic signaling [107] (Figure 10A). However, these findings alone do not establish direct suppression of the mitochondrial apoptotic pathway, as key upstream events, including cyt c release and caspase-9 activation, were not assessed. Further investigation of these mitochondrial apoptotic markers is therefore warranted to determine whether these interventions directly attenuate intrinsic mitochondria-mediated apoptosis in SCA3.

Additionally, erinacine A-enriched mycelial extract counteracts this process by reducing oxidative stress and preserving mitochondrial function, thereby suppressing apoptosis. At the molecular level, erinacine A-enriched mycelial extract downregulates pro-apoptotic factors, including Bax expression, caspase-3/7/9 activation, and poly (ADP-ribose) polymerase (PARP) cleavage, while upregulating the anti-apoptotic protein Bcl-2, restoring the Bcl-2/Bax ratio. In addition, erinacine A-enriched mycelial extract may modulate nuclear factor-κB (NF-κB)- and p53-related apoptotic signaling, which are important regulators of cellular stress and apoptosis. Overall, erinacine A-enriched mycelial extract promotes cell survival by blocking oxidative stress-induced mitochondrial damage and inhibiting the intrinsic mitochondrial-mediated apoptotic pathway in SCA3 models [109]. Remarkably, Wu et al. demonstrated that erinacine A-enriched mycelial extract protects mitochondrial integrity against oxidative stress-induced apoptosis through coordinated antioxidant and stress-response mechanisms. However, whether these effects involve PINK1/Parkin-dependent mitophagy and autophagy–lysosomal degradation, and their contribution to mutant ataxin-3 proteostasis, remains unclear [109].

Similarly, caffeic acid and resveratrol increase Bcl-2 expression in mutant ataxin-3-expressing SK-N-SH-MJD78 cells (Figure 10B) [112]. However, although restoration of Bcl-2 expression and mitochondrial membrane potential was observed, mitochondrial apoptotic signaling, including cytochrome c release and caspase-9 activation, was not directly assessed. Thus, whether caffeic acid and resveratrol attenuate mitochondria-mediated apoptosis in SCA3 remains unclear.

### 7.6. Convergent Mechanisms and Evidence Strength

Across the interventions reviewed, the most consistently reported mitochondrial abnormality is oxidative and redox imbalance, reflected by increased mitochondrial ROS and impaired antioxidant defence. Interventions including AMGZ, licochalcone A, puerarin, daidzein, catalpol, JM17, erinacine A, astragaloside IV, caffeic acid, and resveratrol, reduced ROS and/or enhanced antioxidant defenses, with Nrf2-ARE signaling and SOD2 emerging as recurring components [102,106,107,108,109,110,112]. However, antioxidant activity alone does not necessarily demonstrate functional mitochondrial rescue. Convincing evidence of mitochondrial rescue is provided by interventions that revealed restored mitochondrial membrane potential, respiration, OXPHOS capacity, or mitochondrial biogenesis. In this regard, erinacine A-enriched mycelial extract, astragaloside IV, caffeic acid, and resveratrol restored mitochondrial membrane potential, whereas JM17 and astragaloside IV improved mitochondrial respiration and OXPHOS capacity, and AMGZ and licochalcone A increased PPARGC1A expression associated with mitochondrial biogenesis [102,106,108,109,110,112,175]. Evidence for restoration of mitochondrial dynamics is more limited, with modulation of Drp1/FIS1 and MFN2/OPA1 reported for astragaloside IV in a single SCA3 cellular study [110]. Similarly, direct evidence for suppression of mitochondria-mediated apoptosis is strongest for erinacine A-enriched mycelial extract, which affected Bax, Bcl-2, caspase-9, caspase-3/7, and PARP [109], whereas other interventions showed changes in apoptotic markers without directly assessing key upstream mitochondrial events such as cytochrome c release or caspase-9 activation.

The natural medicines and active compounds were tested predominantly in cellular models, namely human-derived neuronal cell models, including SK-N-SH-MJD78 and SH-SY5Y cells. For instance, PPARGC1A knockdown attenuated the anti-aggregation effects of AMGZ and licochalcone A, while Nrf2 silencing reduced the protective effects of caffeic acid and resveratrol, supporting a functional contribution of these pathways rather than simple associations [106,112]. Causal evidence linking mitochondrial-related pathways to functional outcomes is limited to the SCA3 transgenic *Drosophila* model. A direct link between mitochondrial recovery and functional improvement has not been well established. Overall, the evidence indicates that natural medicines can modulate several interconnected aspects of mitochondrial dysfunction in SCA3, but many observations remain restricted to cellular models, highlighting the need for cautious interpretation of their translational relevance.

## 8. Limitations and Challenges

### 8.1. Batch-to-Batch Variability

The chemical composition and antioxidant activity of natural medicines can vary considerably due to geographical origin, plant genotype, cultivation practices, harvest year, harvest timing, storage conditions, and environmental factors, such as climate and natural habitat [176,177]. These variations can alter the concentration of active compounds, thereby affecting the quality, consistency, and therapeutic efficacy of natural medicines [176,178]. This variability is evident in several compounds. For instance, the composition of cultivation substrates of *H. erinaceus* influences its active properties and metabolite profiles [179,180]. In addition, studies on *A. membranaceus* indicate that both plant age and geographical origin affect its metabolite composition. Older roots contained higher levels of flavonoids, whereas saponins and isoflavonoids served as marker metabolites distinguishing plants from different geographical origins [181].

The chemical complexity of herbal medicines, combined with their widespread use across different markets and regulatory frameworks, further complicates quality control [182]. Advances in chromatographic and spectrometric analyses for unique identification and standardization of natural medicines have been unable to achieve batch-to-batch consistency. Moreover, lack of established sample preparation protocols and validated analytical methods limit the reproducibility of pharmacological and clinical findings [183]. The chemical complexity of herbal medicines, together with variations in raw materials and manufacturing processes, makes quality control particularly challenging [182]. To ensure product identity, purity, and consistency, quality control relies on the authentication of raw materials, standardized cultivation and extraction procedures, and analytical testing of active constituents. Chromatographic fingerprinting techniques, including thin-layer chromatography (TLC), high-performance TLC, and high- performance liquid chromatography, are widely used to detect adulteration and verify product consistency, while international pharmacopoeia guidelines provide standardized approaches for the evaluation of herbal medicines [182,184]. Nevertheless, quality assurance remains limited by the lack of well-defined quality markers, standardized reference materials, and fully validated analytical methods, making consistent batch-to-batch quality difficult to achieve [185].

Natural medicines, mainly being mixtures of various active compounds, require qualitative and quantitative analysis to safeguard their standards, quality, and authenticity [186]. Although chromatographic fingerprinting is currently one of the most powerful, robust, and reliable techniques to assess the batch-to-batch quality of natural medicines, reproducibility remains a challenge [186,187]. Species variation, seasonal and environmental factors, the harvesting process, and the plant parts harvested affect compound quality [186]. In addition, adulteration of the original crude material with synthetically manufactured substances and substandard quality compounds often hinders the quality of active compounds of interest, thus limiting a reliable clinical translation [188].

### 8.2. Pharmacokinetic and Bioavailability

Many natural medicines are characterized by poor aqueous solubility, low bioavailability, and short biological half-life, which collectively limit their therapeutic potential [189,190,191]. These physicochemical limitations reduce absorption, circulation time, and overall drug exposure in the body [192]. Consequently, they lead to reduce in vivo efficacy despite the compounds exhibiting potent activity in vitro [193]. As a result, many natural medicines fail to achieve sufficient drug exposure for effective therapy. Improving formulation and delivery is therefore important to enhance solubility and bioavailability [194].

Importantly, these pharmacokinetic limitations are not uniform across the natural medicines and active compounds reviewed. Their translational relevance depends on compound-specific properties, including absorption, metabolism, systemic exposure, biological half-life, and ability to reach the CNS. Therefore, bioavailability should be considered in relation to the concentrations required to produce the reported mitochondrial effects rather than as a general limitation applicable to all compounds.

The pharmacokinetic studies of AMGZ often present several systemic, physiological, and analytical limitations [195]. These challenges complicate the accurate profiling of its absorption, distribution, metabolism, and excretion pathways. Due to its hydrophilic nature, large molecular weight and chemical structure, AMGZ is poorly absorbed intact across the intestinal epithelium. Additionally, when taken orally, the compound functions predominantly as a prodrug. It undergoes mandatory presystemic hydrolysis by commensal gut microbiota in the large intestine, converting it into glycyrrhetinic acid, an active aglycone metabolite.

Despite exhibiting promising antioxidant and neuroprotective activities, licochalcone A, is limited by its poor oral bioavailability. A pharmacokinetic study in rodents demonstrated poor absolute oral bioavailability of licochalcone A, suggesting inefficient gastrointestinal absorption and extensive first-pass metabolism, resulting in limited systemic exposure following oral administration [196]. Similarly, curcumin exhibits limited therapeutic efficacy because of its poor water solubility, slow absorption, rapid metabolism, and low systemic bioavailability [197]. Following an oral intake, curcumin is rapidly metabolized in the intestine, liver, and kidneys into curcumin glucuronide, curcumin sulfate, and methylated derivatives, which are subsequently excreted in urine and feces [198]. The JM17 is a bioavailable curcumin analogue and should therefore be considered separately from curcumin when evaluating translational exposure.

Additionally, erinacine A had been found to penetrate blood-brain-barrier following an oral administration of erinacine A-enriched mycelial extract in a rodent model. The active compound reached its peak concentration in the brain tissue with a moderate absolute oral bioavailability [199]. These findings provide evidence that constituents of the mycelial extract can reach the brain following systemic administration. Astragaloside IV also exhibits unfavorable pharmacokinetic characteristics. It possesses extremely low oral bioavailability in animal models [200], primarily due to its poor intestinal absorption and low membrane permeability [200,201].

Although puerarin, daidzein, and catalpol can penetrate the blood–brain barrier, potentially exerting neuroprotective effects in the CNS, these active compounds have been reported to show low oral bioavailability and poor water and lipid solubility, which severely limits its clinical pharmacokinetic profile and therapeutic applications [202,203,204]. Puerarin is less distributed in the brain tissue, causing a delayed metabolism. After an oral administration, the gut microbiota can hydrolyze puerarin, and the main products are daidzein and calycosin. Once the concentration of puerarin exceeds a certain value, its metabolism does not increase with increasing substrate concentration, showing obvious saturation. To improve the solubility of puerarin, propylene glycol, ethylene glycol, and polyvinylpyrrolidone have been added to clinical injectable formulations. However, adverse drug reactions may include vascular irritation, fever, allergy and erythrocytolysis after intravenous injection. Likewise, daidzein also shows low systemic bioavailability, rapid phase II metabolism (glucuronidation and sulfation), and high inter-individual variability driven by differences in gut microbiota. Further, catalpol has a short half-life and low oral bioavailability in vivo, preventing broader clinical applications and translational research.

Further, caffeic acid also demonstrates limited exposure following oral administration. In rodents, caffeic acid showed poor absolute bioavailability and intestinal absorption. The reduced systemic availability is associated with weak intestinal permeability and inefficient transport across intestinal epithelial barriers, as demonstrated using Caco-2 cells [205]. Similarly, resveratrol also possesses pharmacokinetic limitations. Although it is readily absorbed by intestinal cells, its systemic availability is greatly reduced due to poor stability, limited solubility, rapid oxidation, and extensive first-pass metabolism. Additionally, resveratrol undergoes extensive phase II metabolism following oral administration, including glucuronidation by UDP-glucuronosyltransferases and sulfation by sulfotransferases, therefore generating conjugated metabolites that substantially reduce circulating levels of free resveratrol [161,206,207].

Collectively, these findings highlight that poor pharmacokinetic properties and lowbioavailability remain major barriers limiting the therapeutic application of natural medicines. However, the extent of this limitation varies among compounds according to their absorption, metabolism, systemic exposure, biological half-life, and CNS penetration. Importantly, concentrations producing mitochondrial effects in SCA3 cellular models cannot always be assumed to be achievable in the brain following systemic administration. Accordingly, pharmacokinetic and pharmacodynamic studies should determine whether therapeutically relevant CNS exposures can be achieved and maintained at doses that provide an acceptable safety margin. Improving formulation and delivery strategies may subsequently help increase systemic and brain exposure where pharmacokinetic limitations restrict therapeutic potential.

### 8.3. Lack of Clinical Trials

Despite promising findings from in vitro and in vivo *studies*, clinical evidence supporting the use of natural medicines for SCA3 remains limited [19]. To date, only one clinical trial (ClinicalTrials.gov ID: NCT05038306) has evaluated *A. membranaceus* in patients with SCA3. In this randomized, triple-blind, placebo-controlled crossover trial, patients received 3 g of concentrated *A. membranaceus* powder twice daily for 12 weeks, followed by a 4-week washout period and crossover to the alternate treatment for a further 12 weeks [208]. Importantly, the study did not assess direct measures of mitochondrial function, such as ATP production, mitochondrial respiration, OXPHOS, mitochondrial membrane potential, ROS, or mitochondrial DNA integrity. Therefore, the observed changes cannot be attributed to mitochondrial rescue. Although the findings suggest potential neuroprotective effects and support the tolerability of *A. membranaceus*, the small sample size and limited clinical evidence restrict the reproducibility of these findings. Larger, adequately powered clinical studies incorporating mitochondrial-related outcomes are needed to determine the efficacy, safety, and underlying mechanisms of natural medicines in SCA3.

Several challenges continue to hinder the clinical translation of natural medicines for SCA3. Current evidence is largely derived from preclinical studies or small Phase 1 and Phase 2 clinical trials that do not fully recapitulate the multisystemic nature of the disease or provide sufficient statistical power to evaluate treatment efficacy [17]. As a result, the therapeutic potential of natural medicines remains largely hypothesis-driven and supported primarily by laboratory-based evidence rather than robust clinical trials incorporating mitochondrial endpoints. In addition, optimal dosing regimens, treatment duration, long-term safety, and pharmacokinetic profiles of natural medicines require further investigation in clinical studies [18]. Therefore, future multicenter, randomized, double-blind, placebo-controlled clinical trials with larger sample sizes and comprehensive assessments of mitochondrial function are essential to validate the therapeutic efficacy and clinical applicability of natural medicines for SCA3.

### 8.4. Potential Toxicity

The relationship between medicinal plants and the human brain, while still relatively unexplored, holds both promise and risks [209]. Although natural medicines are generally considered safer than synthetic drugs, they are not completely free from adverse effects, particularly when administered at high doses or over prolonged periods [210]. In terms of mitochondrial function, some plant compounds, for example, may aggravate ROS production and interfere with mitochondrial homeostasis [209,211].

Several factors influence the safety of plant-derived compounds, including dosage, duration of exposure, formulation, pharmacokinetic properties, and potential herb-drug interactions rather than their natural origin alone. Although numerous active compounds have been shown to exhibit antioxidant and mitochondrial protective effects at therapeutic concentrations, these beneficial properties may become detrimental when administered at high doses, over prolonged periods, or in concentrated formulations, resulting in mitochondrial dysfunction, excessive ROS generation, impaired ATP production, and activation of apoptotic pathways [211,212]. Furthermore, the widespread use of natural medicines has raised concerns regarding their safety because many products are marketed without comprehensive toxicological evaluation or adequate post-marketing surveillance. In many countries, natural medicines are introduced without mandatory safety assessment, standardized manufacturing practices, or stringent quality control, leading to substantial variability in plant species, cultivation conditions, extraction methods, and active constituent efficacy and toxicity [213].

Among the natural medicines discussed in this review, *G. inflata* may cause hypertension, hypokalemia, and fluid retention due to the glycyrrhizin content when consumed in excessive amounts [214]. Caffeic acid has potential toxicity, which is associated with mitochondrial uncoupling, metabolic stress, and cytotoxic effects at higher concentrations, limiting its therapeutic application [215]. Therefore, although these natural medicines are generally well tolerated, careful consideration of dosage, treatment duration, and individual patient characteristics is essential to minimize the risk of adverse effects.

## 9. Future Direction

SCA3 is a complex neurodegenerative disorder involving multiple pathological mechanisms, including mitochondrial dysfunction, oxidative stress, neuroinflammation, and neuronal apoptosis [9]. Mechanistic studies should further clarify the mitochondrial effects of AMGZ and licochalcone A beyond the Nrf2–ARE pathway. Although these compounds activate PPARGC1A and the Nrf2–ARE pathway, reduce oxidative stress, and promote mitochondrial biogenesis, their effects on mitochondrial dynamics, mitophagy, and mitochondrial homeostasis remain unclear. Similarly, although JM17 improves mitochondrial function, reduces mitochondrial ROS, and promotes autophagy-mediated mutant ataxin-3 clearance, its effects on mitochondrial dynamics, mitophagy, and homeostasis remain unclear. Future studies should therefore investigate mitochondrial dynamics, mitophagy, and homeostasis to clarify their contribution to mutant ataxin-3 clearance and cellular protection. Furthermore, erinacine A-enriched mycelial extract attenuates mitochondrial ROS, preserves mitochondrial membrane potential, and suppresses mitochondria-mediated apoptosis in SCA3 models; its effects on mitochondrial bioenergetics, dynamics, biogenesis, and mitophagy remain insufficiently characterized. The specific contribution of erinacine A-enriched mycelial extract to these mitochondrial effects also remains to be established. Astragaloside IV has been shown to improve mitochondrial membrane potential and respiration and reduce excessive mitochondrial fission in SCA3 cells; however, the mechanisms underlying these effects remain unclear. Future studies should therefore clarify whether enhanced antioxidant defense contributes to these mitochondrial benefits and determine the roles of mitochondrial dynamics, mitophagy, and biogenesis in restoring mitochondrial homeostasis. Likewise, although caffeic acid and resveratrol activate Nrf2 signaling, enhance antioxidant defense, restore mitochondrial membrane potential, and promote autophagy, the mechanisms linking Nrf2 activation to mitochondrial protection remain incompletely defined. Their effects on mitochondrial respiration, ATP production, dynamics, mitophagy, and biogenesis warrant further investigation. Moreover, Wu et al. demonstrated restoration of Bcl-2 and mitochondrial membrane potential without directly assessing apoptotic signaling; thus, whether caffeic acid and resveratrol prevent mitochondria-mediated apoptosis in SCA3 remains unclear. Collectively, defining the interplay between antioxidant defense, mitochondrial quality control, and mutant ataxin-3 clearance may clarify the mitochondrial mechanisms underlying the protective effects of these natural medicines [112].

Therefore, targeting a single pathway may not be sufficient to achieve effective disease modification. Future studies should investigate the therapeutic potential of combining natural medicines with complementary mechanisms of action rather than evaluating individual compounds alone [216,217]. Natural medicines may provide enhanced efficacy through synergistic interactions at multiple levels, including molecular targets, disease pathways, cellular processes, and overall therapeutic responses [218]. Moreover, combining active compounds may improve pharmacological activity, solubility, and bioavailability while simultaneously modulating multiple disease-related pathways [219]. Further preclinical and clinical investigations are required to identify effective combinations, optimize dosing regimens, and establish their safety and efficacy in SCA3.

Mesenchymal stem cells (MSCs) represent a promising therapeutic approach for SCA3 and other neurodegenerative disorders due to their immunomodulatory, neuroprotective, and regenerative properties [220]. MSCs are suitable for allogeneic transplantation because of their low immunogenicity, attributed to low major histocompatibility complex (MHC) class I expression and the absence of MHC class II and costimulatory molecules (CD80, CD40, and CD86). At the same time, their secreted cytokines suppress immune cell activation and neuroinflammation [221,222]. In neurological diseases, MSCs may promote tissue repair by secreting neurotrophic growth factors [223], modulating inflammatory responses [224], and releasing extracellular vesicles such as exosomes, which contribute to neuronal survival and functional recovery [225,226]. Under specific induction conditions, human MSCs have also demonstrated neuroglial-like characteristics, including the expression of neuroglial markers and acquisition of functional properties such as resting membrane potential and voltage-sensitive calcium channels, suggesting their potential for neural regeneration [227,228].

Previous studies have shown that preconditioning MSCs with natural medicines can enhance cellular resilience, proliferation, survival, migration, mitochondrial function, and resistance to oxidative stress in other experimental settings [229]. For example, resveratrol preconditioning improves MSC function through activation of the sirtuin 1 (SIRT1)/forkhead box O3 (FOXO3A) signaling pathway [230]. On the other hand, curcumin preconditioning delays cellular senescence and enhances MSC survival by activating autophagy and increasing HO-1 expression, thereby strengthening antioxidant defense and preserving MSC function [229]. These findings raise the possibility that a similar strategy could be explored in SCA3, particularly given the involvement of mitochondrial dysfunction and oxidative stress in SCA3 pathogenesis. However, this proposed application remains a therapeutic hypothesis, as the effects of natural medicine-preconditioned MSCs have not been directly evaluated in SCA3 models. Thus, whether preconditioning can enhance the neuroprotective capacity of MSCs or support neuronal recovery in SCA3 remains to be established. In addition, potential pharmacological antagonism, cumulative toxicity, and altered pharmacokinetics should be considered, as combining natural medicines with MSC-based therapies may not necessarily produce synergistic effects. The therapeutic benefit, safety, and pharmacological compatibility of such combinations therefore require evaluation in disease-relevant SCA3 models.

A major challenge in developing effective SCA3 therapies is the delivery of therapeutic agents to the CNS [231]. Since its pathology primarily affects neurons within the cerebellum, brainstem, and spinal cord, therapeutic molecules must achieve sufficient CNS exposure to exert beneficial effects. However, the blood–brain barrier remains a significant obstacle, as many potentially effective compounds fail to reach the brain after systemic administration [232]. Therefore, future therapeutic strategies should focus on identifying blood–brain barrier-penetrant molecules with suitable physicochemical properties, including optimal molecular size, lipophilicity, and metabolic stability, to enhance CNS delivery [233]. Nanotechnology-based delivery systems have emerged as promising strategies to overcome blood–brain barrier limitations and enhance therapeutic delivery in neurodegenerative diseases, including SCA3. Nanotechnology enables the development of advanced drug delivery platforms that can improve drug absorption, stability, bioavailability, targeting efficiency, and controlled release [234]. Nanoparticles possess unique physicochemical properties, including small size, high surface-area-to-volume ratio, and tunable surface characteristics, which facilitate interactions with biological systems and enhance CNS penetration [235]. Based on their composition, nanoparticles can be categorized into different groups, including lipid-based, polymeric, inorganic, nanocrystalline, and naturally derived nanoparticles, each offering distinct advantages for drug encapsulation, stability, targeting, and therapeutic applications [236,237,238].

Beyond improving general CNS delivery, these platforms could potentially be designed to address specific pharmacokinetic and intracellular barriers identified for the natural medicines reviewed in this study. Lipid nanoparticles could be explored to improve the solubility, stability, and systemic delivery of poorly bioavailable compounds [239], such as licochalcone A, astragaloside IV, caffeic acid, and resveratrol. Biodegradable polymeric nanoparticles could similarly provide controlled release and protect compounds from premature metabolism, potentially prolonging exposure while enabling surface modification for brain-directed delivery [240]. However, these approaches should currently be regarded as prospective strategies for SCA3, as their ability to deliver these specific natural compounds to the SCA3 brain has not been established.

A further opportunity is the development of brain-targeted nanocarriers using surface ligands or other targeting strategies designed to facilitate transport across the blood–brain barrier and increase accumulation within affected neural tissues. This approach may be particularly relevant for compounds that demonstrate mitochondrial activity in vitro but for which sufficient CNS exposure remains uncertain. Importantly, increased brain delivery alone may not ensure mitochondrial protection, because the therapeutic compound must subsequently reach the appropriate intracellular compartment [241]. Thus, mitochondria-targeting moieties, such as mitochondrial-penetrating peptides or other mitochondria-directed ligands, could potentially be incorporated into nanocarriers to facilitate intracellular delivery to mitochondria [242]. Such systems could provide a means of linking the CNS delivery problem with the mitochondrial mechanisms identified in SCA3, although this remains a hypothesis requiring validation in disease-relevant models.

Extracellular vesicle-based delivery represents another prospective approach because extracellular vesicles can naturally transport bioactive molecules and may provide a biocompatible platform for delivering natural compounds or their active constituents to neural cells [243]. Their potential for brain delivery and cellular targeting makes them particularly relevant to neurodegenerative disorders; however, whether extracellular vesicles can effectively deliver the natural medicines reviewed here to mitochondria within SCA3 neurons remains unknown. Future studies should therefore determine whether lipid nanoparticles, biodegradable polymeric nanoparticles, extracellular vesicles, or mitochondria-targeted nanocarriers can improve CNS exposure and intracellular delivery while preserving the biological activity of these natural medicines.

Importantly, evidence from nanotechnology-based delivery systems developed for other neurological or non-SCA3 conditions should not be interpreted as evidence of efficacy in SCA3. Their application to SCA3 remains an extrapolation from broader drug-delivery research and requires validation using SCA3 cellular and animal models. Such studies should establish brain distribution, cellular and mitochondrial localization, pharmacokinetic profiles, dose–response relationships, therapeutic efficacy, and potential toxicity before these approaches can be considered for translational development.

Future studies should focus on developing and optimizing animal models that better replicate the molecular and clinical features of SCA3 to improve the translation of preclinical findings into effective therapies. Although genetically engineered models have contributed to understanding disease mechanisms and evaluating therapeutic candidates, differences between animal models and human disease complexity may limit their predictive value [244]. Therefore, improved disease models and standardized experimental approaches are required to accurately evaluate natural medicines-based therapies targeting oxidative stress, mitochondrial dysfunction, and neurodegeneration before clinical translation. These approaches should consider differences in genetic background, disease stage, tissue vulnerability, and treatment timing, which may contribute to variability in therapeutic outcomes.

While preclinical findings demonstrated the efficacy of natural medicines in restoring mitochondrial function in SCA3, the translation of these findings into a clinical therapeutic strategy remains hypothetical. Human trials are required to determine if these effective concentrations of natural medicines can be safely achieved in human tissues. Collaborative networks among researchers, clinicians, and organizations such as the National Ataxia Foundation (NAF), Ataxia UK, and the Malaysian Rare Disorders Society are essential to promote SCA3 research and accelerate the development of effective therapies. These collaborations may facilitate biomarker discovery, clinical evaluation of emerging treatments, and investigation of complementary therapeutic strategies, including active compounds targeting oxidative stress, mitochondrial dysfunction, and neurodegeneration. Such multidisciplinary efforts could support the translation of promising natural medicines into potential therapeutic approaches for SCA3.

## 10. Conclusions

We provide an in-depth and synthesized evaluation of natural medicines and their associated active compounds that target mitochondrial dysfunction in SCA3. Natural medicines execute their therapeutic effects through attenuation of mitochondrial oxidative stress, enhancement of mitochondrial biogenesis and bioenergetics, preservation of mitochondrial membrane potential and mitochondrial integrity, restoration of mitochondrial dynamics, and suppression of mitochondria-mediated apoptosis. Nevertheless, current evidence is predominantly derived from mechanistic and preclinical studies that do not represent the multisystemic nature of SCA3. The degree to which knowledge translation and the integration of research evidence into clinical practice are aligned and implemented remains unclear. Consolidation of clinical trial methodologies and coordinating regulatory standards are crucial to bridging traditional knowledge with evidence-based medicine and ensuring the global acceptance of natural medicines.

## Figures and Tables

**Table 1:** Natural medicines targeting mitochondrial morphology, function, and homeostasis in SCA3.

Source	Extract and/or Active Compound	Model	Finding	Mechanism of Action	Reference
*Glycyrrhiza inflata*	Ammonium glycyrrhizinate Licochalcone A	Human HEK-293 cells with inducible ATXN3/Q75-GFP expression	↑ Mitochondrial biogenesis, and antioxidant activity ↓ Mitochondrial ROS	Direct ME: ↑ PPARGC1A, SOD2, cyt c Indirect ME: ↑ Nrf2, *HMOX1,* NQO1, GCLC, GSTP1, GSH/GSSG ratio	[106]
*Codonopsis pilosula*	Crude aqueous extract	Human SH-SY5Y-ATXN3/Q75 cells	↓ Apoptosis	Direct ME: ↑ Bcl-2, ↓ Bax Indirect ME: ↓ Caspase-3	[107]
*Ginkgo biloba*					
*Poria cocos*					
*Pueraria lobata*	Crude aqueous extract Puerarin Daidzein	Human SH-SY5Y-ATXN3/Q75 cells	↓ Apoptosis	Direct ME: ↑ Bcl-2, ↓ Bax Indirect ME: ↓ Caspase-3	
		Human HEK-293 ATXN3/Q75-GFP cells	↑ Mitochondrial antioxidant activity ↓ ROS	Direct ME: ↑ SOD2	
*Rehmannia glutinosa*	Crude aqueous extract Catalpol	Human SH-SY5Y-ATXN3/Q75 cells	↓ Apoptosis	Direct ME: ↑ Bcl-2, ↓ Bax Indirect ME: ↓ Caspase-3	
		Human HEK-293 ATXN3/Q75-GFP cells	↑ Mitochondrial antioxidant activity ↓ ROS	Direct ME: ↑ SOD2	
*Curcuma* *longa*	JM17	Human SK-N-SH-MJD78 cells	↑ Mitochondrial respiration, and antioxidant activity ↓ Mitochondrial ROS	Direct ME: ↑ SOD1, SOD2 Indirect ME: ↑ Nrf2, NQO1, HO-1, CAT	[108]
*Hericium erinaceus*	Erinacine A-enriched mycelial extract	Human SK-N-SH-MJD78 cells	↑ Antioxidant activity Restored mitochondrial membrane potential ↓ Mitochondrial ROS, and apoptosis	Direct ME: ↓ Bax, caspase-9, ↑ Bcl-2 Indirect ME: ↓ p53, NF-κB, caspase-7, caspase-3, PARP	[109]
		Transgenic *Drosophila* model (UAS-*MJDtr*-Q78; *elav*-Gal4)	↓ Mitochondrial ROS ↑ Locomotor performance	Direct ME: ↑ Bcl-2, ↓ Bax Indirect ME: ↓ p53, NF-κB	
*Astragalus membranaceus*	Astragaloside IV	Human SK-N-SH-MJD78 cells	↑ Basal and maximal respiration, and OXPHOS Restored mitochondrial membrane potential ↓ Mitochondrial ROS	Direct ME: ↓ Fission proteins (p-Drp1, FIS1) Indirect ME: ↑ SOD	[110]
*Hemerocallis citrina*	Ethanol extract	Transgenic *C. elegans* expressing mutant human ataxin-3 (AT3q130)	↑ Locomotor performance	No effect	[111]
Unknown	Caffeic acid Resveratrol	Human SK-N-SH-MJD78 cells	↑ Mitochondrial antioxidant activity Restored mitochondrial membrane potential ↓ Mitochondrial ROS	Direct ME: ↑ SOD1, SOD2, Bcl-2 Indirect ME: ↑ SIRT1, Nrf2, ↓ HO-1, NQO1, GCLC, GPx1, CAT	[112]

Abbreviations: ATXN3, ataxin-3; Bax, Bcl-2-associated X protein; Bcl-2, B-cell lymphoma 2; CAT, catalase; *C. elegans*, *Caenorhabditis elegans*; cyt c, cytochrome c; FIS1, mitochondrial fission 1 protein; GCLC, glutamate-cysteine ligase catalytic subunit; GFP, Green fluorescent protein; GPx1, glutathione peroxidase 1; GSH, reduced glutathione; GSSG, oxidized glutathione; GSTP1 glutathione S-transferase pi 1; HEK-293, human embryonic kidney-293; *HMOX1*, heme oxygenase 1; HO-1, heme oxygenase 1; JM17, curcumin analog; MJD, Machado-Joseph disease; ME, mitochondrial effect; NQO1, NAD(P)H quinone dehydrogenase 1; NF-κB, nuclear factor-κB; Nrf2, NF-E2-related factor 2; OXPHOS, oxidative phosphorylation; PARP, poly (ADP-ribose) polymerase; p-Drp1, phosphorylated dynamin-related protein 1; PPARGC1A, peroxisome proliferator-activated receptor gamma coactivator 1-alpha; ROS, reactive oxygen species; SCA3, spinocerebellar ataxia type 3; SIRT1, sirtuin 1; SH-SY5Y-ATXN3/Q75, human neuroblastoma cells expressing ATXN3/Q75-GFP; SK-N-SH-MJD78, human neuroblastoma cells transfected with full-length MJD with 78 CAG repeats; SOD, superoxide dismutase; SOD1, superoxide dismutase 1; SOD2, superoxide dismutase 2.

## Data Availability

The original contributions presented in this study are included in the article. Further inquiries can be directed to the corresponding authors.

## References

[1] Sullivan R., Yau W.Y., O’Connor E., Houlden H. (2019). Spinocerebellar Ataxia: An Update. J. Neurol..

[2] Silva A., de Almeida A.V., Macedo-Ribeiro S. (2018). Polyglutamine Expansion Diseases: More than Simple Repeats. J. Struct. Biol..

[3] Stahl F., Evert B.O., Han X., Breuer P., Wüllner U. (2024). Spinocerebellar Ataxia Type 3 Pathophysiology—Implications for Translational Research and Clinical Studies. Int. J. Mol. Sci..

[4] Paulson H., Shakkottai V., Adam M.P., Bick S., Mirzaa G.M., Pagon R.A., Wallace S.E., Amemiya A. (1993). Spinocerebellar Ataxia Type 3. GeneReviews^®^.

[5] Bhandari J., Thada P.K., Samanta D. (2023). Spinocerebellar Ataxia. StatPearls.

[6] Bettencourt C., Lima M. (2011). Machado-Joseph Disease: From First Descriptions to New Perspectives. Orphanet J. Rare Dis..

[7] van Prooije T., Ibrahim N.M., Azmin S., van de Warrenburg B. (2021). Spinocerebellar Ataxias in Asia: Prevalence, Phenotypes and Management. Park. Relat. Disord..

[8] Mohamed Ibrahim N., Lau Y.H., Ariffin N., Md Desa S.H., Azizan E., Chin L.K., Md Rani S.A., Yakob Y., Datuk Puvanarajah S., van de Warrenburg B. (2020). Frequency of Spinocerebellar Ataxia Type 1, 2, 3,6 and 7 and Clinical Profile of Spinocerebellar Ataxia Type 3 in Malaysia. Cerebellum Ataxias.

[9] Mohd Hisam N.S., Wong K.H. (2024). Oxidative Stress in Spinocerebellar Ataxia Type 3 and Its Attenuation by Herbal Remedies in Traditional Chinese Medicine: A Systematic Review. Antioxidants.

[10] Evers M.M., Toonen L.J.A., van Roon-Mom W.M.C. (2014). Ataxin-3 Protein and RNA Toxicity in Spinocerebellar Ataxia Type 3: Current Insights and Emerging Therapeutic Strategies. Mol. Neurobiol..

[11] Raj K., Akundi R.S. (2021). Mutant Ataxin-3-Containing Aggregates (MATAGGs) in Spinocerebellar Ataxia Type 3: Dynamics of the Disorder. Mol. Neurobiol..

[12] Wang Z. (2018). Experimental and Clinical Strategies for Treating Spinocerebellar Ataxia Type 3. Neuroscience.

[13] Cocozza S. (2021). Editorial: The Cerebellum: From Vascular Disease to Neurodegeneration. Front. Neurol..

[14] Hsu J.-Y., Jhang Y.-L., Cheng P.-H., Chang Y.-F., Mao S.-H., Yang H.-I., Lin C.-W., Chen C.-M., Yang S.-H. (2017). The Truncated C-Terminal Fragment of Mutant ATXN3 Disrupts Mitochondria Dynamics in Spinocerebellar Ataxia Type 3 Models. Front. Mol. Neurosci..

[15] Berger M., Garcia-Moreno H., Ferreira M., Hubener-Schmid J., Schaprian T., Wegner P., Elter T., Teichmann K.M., Santana M.M., Grobe-Einsler M. (2025). Progression of Biological Markers in Spinocerebellar Ataxia Type 3: Longitudinal Analysis of Prospective Data from the ESMI Cohort. Lancet Reg. Health-Eur..

[16] Naveed M., Ali N., Aziz T., Hanif N., Fatima M., Ali I., Alharbi M., Alasmari A.F., Albekairi T.H. (2024). The Natural Breakthrough: Phytochemicals as Potent Therapeutic Agents against Spinocerebellar Ataxia Type 3. Sci. Rep..

[17] Lew S.Y., Phang M.W.L., Chong P.S., Roy J., Poon C.H., Yu W.S., Lim L.W., Wong K.H. (2022). Discovery of Therapeutics Targeting Oxidative Stress in Autosomal Recessive Cerebellar Ataxia: A Systematic Review. Pharmaceuticals.

[18] Phang M.W.L., Lew S.Y., Chung I., Lim W.K.-S., Lim L.W., Wong K.H. (2021). Therapeutic Roles of Natural Remedies in Combating Hereditary Ataxia: A Systematic Review. Chin. Med..

[19] Roney M., Mohd Hisam N.S., Uddin M.N., Istiaque Hamim S.M., Lim L.W., Wong K.H. (2026). Repurposing of Natural Products for Spinocerebellar Ataxia Type 3 Using Integrated Network Pharmacology and in Silico Approaches. Sci. Rep..

[20] Wu Y.-L., Sun H.-L., Chang J.-C., Lin W.-Y., Chen P.-Y., Chen C.-C., Lee L.-Y., Li C.-C., Hsieh M., Chen H.-W. (2024). Erinacine A-Enriched *Hericium erinaceus* Mycelium Ethanol Extract Lessens Cellular Damage in Cell and Drosophila Models of Spinocerebellar Ataxia Type 3 by Improvement of Nrf2 Activation. Antioxidants.

[21] Cui Z.-T., Mao Z.-T., Yang R., Li J.-J., Jia S.-S., Zhao J.-L., Zhong F.-T., Yu P., Dong M. (2024). Spinocerebellar Ataxias: From Pathogenesis to Recent Therapeutic Advances. Front. Neurosci..

[22] Chen Y., Li D., Wei M., Zhou M., Zhang L., Yu J., Qiu M., Jin Y., Lu X. (2021). Homozygous Spinocerebellar Ataxia Type 3 in China: A Case Report. J. Int. Med. Res..

[23] Schuster K.H., Putka A.F., McLoughlin H.S. (2022). Pathogenetic Mechanisms Underlying Spinocerebellar Ataxia Type 3 Are Altered in Primary Oligodendrocyte Culture. Cells.

[24] McLoughlin H.S., Moore L.R., Paulson H.L. (2020). Pathogenesis of SCA3 and Implications for Other Polyglutamine Diseases. Neurobiol. Dis..

[25] Kourkouta E., Weij R., González-Barriga A., Mulder M., Verheul R., Bosgra S., Groenendaal B., Puoliväli J., Toivanen J., van Deutekom J.C.T. (2019). Suppression of Mutant Protein Expression in SCA3 and SCA1 Mice Using a CAG Repeat-Targeting Antisense Oligonucleotide. Mol. Ther. Nucleic Acids.

[26] Johnson S.L., Ranxhi B., Libohova K., Tsou W.-L., Todi S.V. (2020). Ubiquitin-Interacting Motifs of Ataxin-3 Regulate Its Polyglutamine Toxicity through Hsc70-4-Dependent Aggregation. eLife.

[27] Weishäupl D., Schneider J., Peixoto Pinheiro B., Ruess C., Dold S.M., von Zweydorf F., Gloeckner C.J., Schmidt J., Riess O., Schmidt T. (2019). Physiological and Pathophysiological Characteristics of Ataxin-3 Isoforms. J. Biol. Chem..

[28] Hernández-Carralero E., Quinet G., Freire R. (2024). ATXN3: A Multifunctional Protein Involved in the Polyglutamine Disease Spinocerebellar Ataxia Type 3. Expert Rev. Mol. Med..

[29] Rodrigues A.-J., Coppola G., Santos C., Costa M.d.C., Ailion M., Sequeiros J., Geschwind D.H., Mariel P. (2007). Functional Genomics and Biochemical Characterization of the *C. elegans* Orthologue of the Machado-Joseph Disease Protein Ataxin-3. FASEB J..

[30] Schmitt I., Linden M., Khazneh H., Evert B.O., Breuer P., Klockgether T., Wuellner U. (2007). Inactivation of the Mouse *Atxn3* (Ataxin-3) Gene Increases Protein Ubiquitination. Biochem. Biophys. Res. Commun..

[31] Ashkenazi A., Bento C.F., Ricketts T., Vicinanza M., Siddiqi F., Pavel M., Squitieri F., Hardenberg M.C., Imarisio S., Menzies F.M. (2017). Polyglutamine Tracts Regulate Autophagy. Autophagy.

[32] Yue H.-W., Hong J.-Y., Zhang S.-X., Jiang L.-L., Hu H.-Y. (2021). PolyQ-Expanded Proteins Impair Cellular Proteostasis of Ataxin-3 through Sequestering the Co-Chaperone HSJ1 into Aggregates. Sci. Rep..

[33] Li L.-B., Yu Z., Teng X., Bonini N.M. (2008). RNA Toxicity Is a Component of Ataxin-3 Degeneration in Drosophila. Nature.

[34] Wang L.-C., Chen K.-Y., Pan H., Wu C.-C., Chen P.-H., Liao Y.-T., Li C., Huang M.-L., Hsiao K.-M. (2010). Muscleblind Participates in RNA Toxicity of Expanded CAG and CUG Repeats in *Caenorhabditis elegans*. Cell Mol. Life Sci..

[35] Cleary J.D., Pattamatta A., Ranum L.P.W. (2018). Repeat-Associated Non-ATG (RAN) Translation. J. Biol. Chem..

[36] Jazurek-Ciesiolka M., Ciesiolka A., Komur A.A., Urbanek-Trzeciak M.O., Krzyzosiak W.J., Fiszer A. (2020). RAN Translation of the Expanded CAG Repeats in the SCA3 Disease Context. J. Mol. Biol..

[37] Lin Y., Wilson J.H. (2011). Transcription-Induced DNA Toxicity at Trinucleotide Repeats. Cell Cycle.

[38] Wojciechowska M., Krzyzosiak W.J. (2011). CAG Repeat RNA as an Auxiliary Toxic Agent in Polyglutamine Disorders. RNA Biol..

[39] Lin Y., Leng M., Wan M., Wilson J.H. (2010). Convergent Transcription through a Long CAG Tract Destabilizes Repeats and Induces Apoptosis. Mol. Cell Biol..

[40] Zeng L., Zhang D., McLoughlin H.S., Zalon A.J., Aravind L., Paulson H.L. (2018). Loss of the Spinocerebellar Ataxia Type 3 Disease Protein ATXN3 Alters Transcription of Multiple Signal Transduction Pathways. PLoS ONE.

[41] Hernández-Carralero E., Cabrera E., Rodríguez-Torres G., Hernández-Reyes Y., Singh A.N., Santa-María C., Fernández-Justel J.M., Janssens R.C., Marteijn J.A., Evert B.O. (2023). ATXN3 Controls DNA Replication and Transcription by Regulating Chromatin Structure. Nucleic Acids Res..

[42] Chou A.-H., Yeh T.-H., Ouyang P., Chen Y.-L., Chen S.-Y., Wang H.-L. (2008). Polyglutamine-Expanded Ataxin-3 Causes Cerebellar Dysfunction of SCA3 Transgenic Mice by Inducing Transcriptional Dysregulation. Neurobiol. Dis..

[43] Nalavade R., Griesche N., Ryan D.P., Hildebrand S., Krauß S. (2013). Mechanisms of RNA-Induced Toxicity in CAG Repeat Disorders. Cell Death Dis..

[44] Tsoi H., Lau T.C.-K., Tsang S.-Y., Lau K.-F., Chan H.Y.E. (2012). CAG Expansion Induces Nucleolar Stress in Polyglutamine Diseases. Proc. Natl. Acad. Sci. USA.

[45] Tsoi H., Chan H.Y.E. (2013). Expression of Expanded CAG Transcripts Triggers Nucleolar Stress in Huntington’s Disease. Cerebellum.

[46] Moreira-Gomes T., Nóbrega C. (2024). From the Disruption of RNA Metabolism to the Targeting of RNA-Binding Proteins: The Case of Polyglutamine Spinocerebellar Ataxias. J. Neurochem..

[47] Andersen J.V. (2025). The Glutamate/GABA-Glutamine Cycle: Insights, Updates, and Advances. J. Neurochem..

[48] Magi S., Piccirillo S., Amoroso S., Lariccia V. (2019). Excitatory Amino Acid Transporters (EAATs): Glutamate Transport and Beyond. Int. J. Mol. Sci..

[49] Zhou Y., Danbolt N.C. (2014). Glutamate as a Neurotransmitter in the Healthy Brain. J. Neural Transm..

[50] Magi S., Piccirillo S., Amoroso S. (2019). The Dual Face of Glutamate: From a Neurotoxin to a Potential Survival Factor—Metabolic Implications in Health and Disease. Cell Mol. Life Sci..

[51] Power E.M., Empson R.M. (2014). Functional Contributions of Glutamate Transporters at the Parallel Fibre to Purkinje Neuron Synapse–Relevance for the Progression of Cerebellar Ataxia. Cerebellum Ataxias.

[52] Cellot G., Ballerini L. (2025). Interplay Among Synaptic Glutamate Release and Excitotoxicity: Neuronal Damage and Graphene-Based Materials Related Protection. Life.

[53] Karki P., Hong P., Johnson J., Pajarillo E., Son D.-S., Aschner M., Lee E.Y. (2018). Arundic Acid Increases Expression and Function of Astrocytic Glutamate Transporter EAAT1 Via the ERK, Akt, and NF-κB Pathways. Mol. Neurobiol..

[54] Magdaleno Roman J.Y., Chapa González C. (2024). Glutamate and Excitotoxicity in Central Nervous System Disorders: Ionotropic Glutamate Receptors as a Target for Neuroprotection. Neuroprotection.

[55] Chuang C.-Y., Yang C.-C., Soong B.-W., Yu C.-Y., Chen S.-H., Huang H.-P., Kuo H.-C. (2019). Modeling Spinocerebellar Ataxias 2 and 3 with iPSCs Reveals a Role for Glutamate in Disease Pathology. Sci. Rep..

[56] Nóbrega C., Marcelo A., Rajado A.T., Conceição A., Estevam B., Matos C.A., Brito D.V.C., Afonso I.T., Codêsso J.M., Koppenol R. (2025). Molecular Hallmarks of Neurodegeneration in Polyglutamine Spinocerebellar Ataxias. Cell Death Dis..

[57] Kristensen L.V., Oppermann F.S., Rauen M.J., Fog K., Schmidt T., Schmidt J., Harmuth T., Hartmann-Petersen R., Thirstrup K. (2018). Mass Spectrometry Analyses of Normal and Polyglutamine Expanded Ataxin-3 Reveal Novel Interaction Partners Involved in Mitochondrial Function. Neurochem. Int..

[58] Joshi D.C., Chavan M.B., Gurow K., Gupta M., Dhaliwal J.S., Ming L.C. (2025). The Role of Mitochondrial Dysfunction in Huntington’s Disease: Implications for Therapeutic Targeting. Biomed. Pharmacother..

[59] Vieira J., Barros M., López-Fernández H., Glez-Peña D., Nogueira-Rodríguez A., Vieira C.P. (2025). Predicting Which Mitophagy Proteins Are Dysregulated in Spinocerebellar Ataxia Type 3 (SCA3) Using the Auto-P2docking Pipeline. Int. J. Mol. Sci..

[60] Bonanomi M., Visentin C., Invernizzi G., Tortora P., Regonesi M.E. (2015). The Toxic Effects of Pathogenic Ataxin-3 Variants in a Yeast Cellular Model. PLoS ONE.

[61] Harmuth T., Weber J.J., Zimmer A.J., Sowa A.S., Schmidt J., Fitzgerald J.C., Schöls L., Riess O., Hübener-Schmid J. (2022). Mitochondrial Dysfunction in Spinocerebellar Ataxia Type 3 Is Linked to VDAC1 Deubiquitination. Int. J. Mol. Sci..

[62] Harmuth T., Prell-Schicker C., Weber J.J., Gellerich F., Funke C., Drießen S., Magg J.C.D., Krebiehl G., Wolburg H., Hayer S.N. (2018). Mitochondrial Morphology, Function and Homeostasis Are Impaired by Expression of an N-Terminal Calpain Cleavage Fragment of Ataxin-3. Front. Mol. Neurosci..

[63] Costa V., Giacomello M., Hudec R., Lopreiato R., Ermak G., Lim D., Malorni W., Davies K.J.A., Carafoli E., Scorrano L. (2010). Mitochondrial Fission and Cristae Disruption Increase the Response of Cell Models of Huntington’s Disease to Apoptotic Stimuli. EMBO Mol. Med..

[64] De Moura M.B., Dos Santos L.S., Van Houten B. (2010). Mitochondrial Dysfunction in Neurodegenerative Diseases and Cancer. Environ. Mol. Mutagen..

[65] Kazachkova N., Raposo M., Montiel R., Cymbron T., Bettencourt C., Silva-Fernandes A., Silva S., Maciel P., Lima M. (2013). Patterns of Mitochondrial DNA Damage in Blood and Brain Tissues of a Transgenic Mouse Model of Machado-Joseph Disease. Neurodegener. Dis..

[66] Piasecki P., Wiatr K., Ruszkowski M., Marczak Ł., Trottier Y., Figiel M. (2023). Impaired Interactions of Ataxin-3 with Protein Complexes Reveals Their Specific Structure and Functions in SCA3 Ki150 Model. Front. Mol. Neurosci..

[67] Chen W., Zhao H., Li Y. (2023). Mitochondrial Dynamics in Health and Disease: Mechanisms and Potential Targets. Signal Transduct. Target. Ther..

[68] Chou A.-H., Yeh T.-H., Kuo Y.-L., Kao Y.-C., Jou M.-J., Hsu C.-Y., Tsai S.-R., Kakizuka A., Wang H.-L. (2006). Polyglutamine-Expanded Ataxin-3 Activates Mitochondrial Apoptotic Pathway by Upregulating Bax and Downregulating Bcl-xL. Neurobiol. Dis..

[69] Almeida F., Ferreira I.L., Naia L., Marinho D., Vilaça-Ferreira A.C., Costa M.D., Duarte-Silva S., Maciel P., Rego A.C. (2023). Mitochondrial Dysfunction and Decreased Cytochrome c in Cell and Animal Models of Machado–Joseph Disease. Cells.

[70] Durcan T.M., Kontogiannea M., Thorarinsdottir T., Fallon L., Williams A.J., Djarmati A., Fantaneanu T., Paulson H.L., Fon E.A. (2011). The Machado–Joseph Disease-Associated Mutant Form of Ataxin-3 Regulates Parkin Ubiquitination and Stability. Hum. Mol. Genet..

[71] Paulino L.A., Martins A.P., França Júnior M.C. (2026). Managing Symptoms and Improving the Quality of Life of Persons with Machado-Joseph Disease. Expert Rev. Neurother..

[72] Ghanekar S.D., Kuo S.-H., Staffetti J.S., Zesiewicz T.A. (2022). Current and Emerging Treatment Modalities for Spinocerebellar Ataxias. Expert Rev. Neurother..

[73] Dhuri K., Bechtold C., Quijano E., Pham H., Gupta A., Vikram A., Bahal R. (2020). Antisense Oligonucleotides: An Emerging Area in Drug Discovery and Development. J. Clin. Med..

[74] Potashman M.H., Popoff E., Powell L.C., Beiner M.W., Mackenzie A., Coric V., Subramony S., Synofzik M., Schmahmann J., L’Italien G. (2025). Measurement Properties of the Friedreich Ataxia Rating Scale in Patients with Spinocerebellar Ataxia. Neurol. Ther..

[75] Chen Y.-S., Hong Z.-X., Lin S.-Z., Harn H.-J. (2020). Identifying Therapeutic Targets for Spinocerebellar Ataxia Type 3/Machado–Joseph Disease through Integration of Pathological Biomarkers and Therapeutic Strategies. Int. J. Mol. Sci..

[76] Svenningsson P., Odin P., Bergquist F., Wirdefeldt K., Nyholm D., Andréasson M., Markaki I., Johansson A.C., Jergil M., Jankosky C. (2025). NLX-112 Randomized Phase 2A Trial: Safety, Tolerability, Anti-Dyskinetic, and Anti-Parkinsonian Efficacy. Mov. Disord..

[77] Pereira-Sousa J., Ferreira-Lomba B., Bellver-Sanchis A., Vilasboas-Campos D., Fernandes J.H., Costa M.D., Varney M.A., Newman-Tancredi A., Maciel P., Teixeira-Castro A. (2021). Identification of the 5-HT1A Serotonin Receptor as a Novel Therapeutic Target in a *C. elegans* Model of Machado-Joseph Disease. Neurobiol. Dis..

[78] Ferreira-Lomba B., Guerreiro S., Duarte-Silva S., Cunha-Garcia D., Oliveira S., Vieira C., Pereira-Sousa J., Vilasboas-Campos D., Vidinha-Mira A., Monteiro-Fernandes D. (2025). Efficacy of Chronic 5-HT1A Receptor Agonism by NLX-112 in a Mouse Model of Spinocerebellar Ataxia Type 3. bioRxiv.

[79] Vico Therapeutics B. V. (2025). A Phase 1/2a, Open-Label Trial to Investigate the Safety, Tolerability, Pharmacokinetics and Pharmacodynamics of Multiple Ascending Doses of Intrathecally Administered VO659 in Participants with Spinocerebellar Ataxia Types 1, 3 and Huntington’s Disease.

[80] Schmidt J., Schmidt T., Golla M., Lehmann L., Weber J.J., Hübener-Schmid J., Riess O. (2016). In Vivo Assessment of Riluzole as a Potential Therapeutic Drug for Spinocerebellar Ataxia Type 3. J. Neurochem..

[81] L’Italien G.J., Oikonomou E.K., Khera R., Potashman M.H., Beiner M.W., Maclaine G.D.H., Schmahmann J.D., Perlman S., Coric V. (2024). Video-Based Kinematic Analysis of Movement Quality in a Phase 3 Clinical Trial of Troriluzole in Adults with Spinocerebellar Ataxia: A Post Hoc Analysis. Neurol. Ther..

[82] Biohaven Pharmaceuticals, Inc. (2025). A Phase III, Long-Term, Randomized, Double-Blind, Placebo-Controlled Trial of Troriluzole in Adult Participants with Spinocerebellar Ataxia.

[83] Lucas S. (2025). NAF Statement on FDA Issuing Complete Response Letter for VYGLXIA (Troriluzole).

[84] Pohl F.K.T., Lin P. (2018). The Potential Use of Plant Natural Products and Plant Extracts with Antioxidant Properties for the Prevention/Treatment of Neurodegenerative Diseases: In Vitro, In Vivo and Clinical Trials. Molecules.

[85] Jin H., Aquili L., Heng B.C., Strekalova T., Fung M.-L., Tipoe G.L., Kocabicak E., Blokland A., Temel Y., Lu Z. (2025). *Ganoderma lucidum*: An Emerging Nutritional Approach to Manage Depression. Food Rev. Int..

[86] Lew S.Y., Lim S.H., Lim L.W., Wong K.H. (2020). Neuroprotective Effects of *Hericium erinaceus* (Bull.: Fr.) Pers. against High-Dose Corticosterone-Induced Oxidative Stress in PC-12 Cells. BMC Complement. Med. Ther..

[87] Ngu E.-L., Tan C.-Y., Lai N.J.-Y., Wong K.-H., Lim S.-H., Ming L.C., Tan K.-O., Phang S.-M., Yow Y.-Y. (2022). *Spirulina platensis* Suppressed iNOS and Proinflammatory Cytokines in Lipopolysaccharide-Induced BV2 Microglia. Metabolites.

[88] Pang J.-R., Goh V.M.-J., Tan C.-Y., Phang S.-M., Wong K.-H., Yow Y.-Y. (2018). Neuritogenic and in Vitro Antioxidant Activities of Malaysian *Gracilaria manilaensis* Yamamoto & Trono. J. Appl. Phycol..

[89] Phan C.-W., David P., Wong K.-H., Naidu M., Sabaratnam V. (2015). Uridine from *Pleurotus giganteus* and Its Neurite Outgrowth Stimulatory Effects with Underlying Mechanism. PLoS ONE.

[90] Phang M.W.L., Hisam N.S.M., Supandi F., Cheng P.G., Lim S.H., Lim L.W., Wong K.H. (2025). The Tiger Milk Medicinal Mushroom *Lignosus rhinocerus* (Agaricomycetes) Mitigates Oxidative Damage in a Cellular Model Mimicking Friedreich’s Ataxia. Int. J. Med. Mushrooms.

[91] Subermaniam K., Yow Y.Y., Lim S.H., Koh O.H., Wong K.H. (2020). Malaysian Macroalga *Padina australis* Hauck Attenuates High Dose Corticosterone-Mediated Oxidative Damage in PC12 Cells Mimicking the Effects of Depression. Saudi J. Biol. Sci..

[92] Subermaniam K., Teoh S.L., Yow Y.-Y., Tang Y.Q., Lim L.W., Wong K.H. (2021). Marine Algae as Emerging Therapeutic Alternatives for Depression: A Review. Iran. J. Basic Med. Sci..

[93] Subermaniam K., Lew S.Y., Yow Y.Y., Lim S.H., Yu W.S., Lim L.W., Wong K.H. (2023). Malaysian Brown Macroalga *Padina australis* Mitigates Lipopolysaccharide-Stimulated Neuroinflammation in BV2 Microglial Cells. Iran. J. Basic Med. Sci..

[94] Wong K.-H., Ng C.-C., Kanagasabapathy G., Yow Y.-Y., Sabaratnam V. (2017). An Overview of Culinary and Medicinal Mushrooms in Neurodegeneration and Neurotrauma Research. Int. J. Med. Mushrooms.

[95] Woon C.K., Hui W.K., Abas R., Haron M.H., Das S., Lin T.S. (2022). Natural Product-Based Nanomedicine: Recent Advances and Issues for the Treatment of Alzheimer’s Disease. Curr. Neuropharmacol..

[96] World Health Organization WHO Releases Guidelines on Chronic Low Back Pain. https://www.who.int/news/item/07-12-2023-who-releases-guidelines-on-chronic-low-back-pain.

[97] Kim M.R. (2021). Antioxidants of Natural Products. Antioxidants.

[98] Nahar L., Charoensup R., Kalieva K., Habibi E., Guo M., Wang D., Kvasnica M., Onder A., Sarker S.D. (2025). Natural Products in Neurodegenerative Diseases: Recent Advances and Future Outlook. Front. Pharmacol..

[99] Chen S., Wang M., Veeraperumal S., Teng B., Li R., Qian Z., Chen J., Zhong S., Cheong K.-L. (2023). Antioxidative and Protective Effect of *Morchella esculenta* against Dextran Sulfate Sodium-Induced Alterations in Liver. Foods.

[100] Chau S.C., Chong P.S., Jin H., Tsui K.C., Khairuddin S., Tse A.C.K., Lew S.Y., Tipoe G.L., Lee C.W., Fung M.-L. (2023). *Hericium erinaceus* Promotes Anti-Inflammatory Effects and Regulation of Metabolites in an Animal Model of Cerebellar Ataxia. Int. J. Mol. Sci..

[101] Lew S.-Y., Yow Y.-Y., Lim L.-W., Wong K.-H. (2020). Antioxidant-Mediated Protective Role of *Hericium erinaceus* (Bull.: Fr.) Pers. against Oxidative Damage in Fibroblasts from Friedreich’s Ataxia Patient. Food Sci. Technol..

[102] Zong Y., Li H., Liao P., Chen L., Pan Y., Zheng Y., Zhang C., Liu D., Zheng M., Gao J. (2024). Mitochondrial Dysfunction: Mechanisms and Advances in Therapy. Signal Transduct. Target. Ther..

[103] Harrington J.S., Ryter S.W., Plataki M., Price D.R., Choi A.M.K. (2023). Mitochondria in Health, Disease, and Aging. Physiol. Rev..

[104] Wadan A.-H.S., Shaaban A.H., El-Sadek M.Z., Mostafa S.A., Moshref A.S., El-Hussein A., Ellakwa D.E.-S., Mehanny S.S. (2025). Mitochondrial-Based Therapies for Neurodegenerative Diseases: A Review of the Current Literature. Naunyn Schmiedebergs Arch. Pharmacol..

[105] Das S., Zea M.P., Russon M.P., Xing Z., Torregrosa-Allen S., Cervantes H.E., Harper H.A., Elzey B.D., Tran E.J. (2025). Supinoxin Blocks Small Cell Lung Cancer Progression by Inhibiting Mitochondrial Respiration through DDX5. iScience.

[106] Chen C.-M., Weng Y.-T., Chen W.-L., Lin T.-H., Chao C.-Y., Lin C.-H., Chen I.-C., Lee L.-C., Lin H.-Y., Wu Y.-R. (2014). Aqueous Extract of *Glycyrrhiza inflata* Inhibits Aggregation by Upregulating PPARGC1A and NFE2L2–ARE Pathways in Cell Models of Spinocerebellar Ataxia 3. Free Radic. Biol. Med..

[107] Chen I.-C., Chang C.-N., Chen W.-L., Lin T.-H., Chao C.-Y., Lin C.-H., Lin H.-Y., Cheng M.-L., Chiang M.-C., Lin J.-Y. (2019). Targeting Ubiquitin Proteasome Pathway with Traditional Chinese Medicine for Treatment of Spinocerebellar Ataxia Type 3. Am. J. Chin. Med..

[108] Wu Y.-L., Chang J.-C., Chao Y.-C., Chan H., Hsieh M., Liu C.-S. (2022). In Vitro Efficacy and Molecular Mechanism of Curcumin Analog in Pathological Regulation of Spinocerebellar Ataxia Type 3. Antioxidants.

[109] Wu Y.-L., Chen S.-C., Chang J.-C., Lin W.-Y., Chen C.-C., Li C.-C., Hsieh M., Chen H.-W., Chang T.-Y., Liu C.-S. (2023). The Protective Effect of Erinacine A–Enriched *Hericium erinaceus* Mycelium Ethanol Extract on Oxidative Stress–Induced Neurotoxicity in Cell and Drosophila Models of Spinocerebellar Ataxia Type 3. Free Radic. Biol. Med..

[110] Lin Y., Cheng W., Chang J., Wu Y., Hsieh M., Liu C. (2024). Astragaloside IV Reduces Mutant Ataxin-3 Levels and Supports Mitochondrial Function in Spinocerebellar Ataxia Type 3. Sci. Rep..

[111] Fernandes J.H., Costa M.D., Vilasboas-Campos D., Ferreira-Lomba B., Pereira-Sousa J., Wang Q., Teixeira-Castro A., Liu X., Wang F., Dias A.C.P. (2025). Therapeutic Effects of *Hemerocallis citrina* Baroni Extract on Animal Models of Neurodegenerative Diseases Through Serotonin and HLH-30/TFEB-Dependent Mechanisms. Int. J. Mol. Sci..

[112] Wu Y.-L., Chang J.-C., Lin W.-Y., Li C.-C., Hsieh M., Chen H.-W., Wang T.-S., Wu W.-T., Liu C.-S., Liu K.-L. (2018). Caffeic Acid and Resveratrol Ameliorate Cellular Damage in Cell and Drosophila Models of Spinocerebellar Ataxia Type 3 through Upregulation of Nrf2 Pathway. Free Radic. Biol. Med..

[113] Zeng J., Huang Y., Zhou L., Liang X., Yang C., Wang H., Yuan L., Wang Y., Li Y. (2023). Histone Deacetylase GiSRT2 Negatively Regulates Flavonoid Biosynthesis in *Glycyrrhiza inflata*. Cells.

[114] Lim T.K. (2016). *Glycyrrhiza glabra*. Edible Medicinal and Non-Medicinal Plants.

[115] Raman V., Manfron J., Avula B., Zhao J., Katragunta K., Chittiboyina A.G., Khan I.A. (2022). Application of Microscopy in the Quality Control of Licorice Roots: Comparative Anatomy of the Roots and Rhizomes of Five Species of Glycyrrhiza. Microsc. Microanal..

[116] Kolbe L., Immeyer J., Batzer J., Wensorra U., Tom Dieck K., Mundt C., Wolber R., Stäb F., Schönrock U., Ceilley R.I. (2006). Anti-Inflammatory Efficacy of Licochalcone A: Correlation of Clinical Potency and in Vitro Effects. Arch. Dermatol. Res..

[117] Liu J., Luo L., Zhang H., Jia B., Lu J., Li P., Chen J. (2015). Rapid Screening for Novel Antioxidants in *Glycyrrhiza inflata* Using High-Resolution Peak Fractionation. J. Funct. Foods.

[118] Tang Z., Wang F., Lv J., Yang G., Shen X., Zeng F., Meng L., Zhou W., Zhan L., Weng Z. (2025). Neuroprotective Effect of Licochalcone A against Aluminum Chloride-Induced Neurotoxicity by Reducing Aβ Accumulation, Oxidative Stress and Inflammatory Reaction. NeuroToxicology.

[119] Maione F., Minosi P., Di Giannuario A., Raucci F., Chini M.G., De Vita S., Bifulco G., Mascolo N., Pieretti S. (2019). Long-Lasting Anti-Inflammatory and Antinociceptive Effects of Acute Ammonium Glycyrrhizinate Administration: Pharmacological, Biochemical, and Docking Studies. Molecules.

[120] Ciarlo L., Marzoli F., Minosi P., Matarrese P., Pieretti S. (2021). Ammonium Glycyrrhizinate Prevents Apoptosis and Mitochondrial Dysfunction Induced by High Glucose in SH-SY5Y Cell Line and Counteracts Neuropathic Pain in Streptozotocin-Induced Diabetic Mice. Biomedicines.

[121] Kocaadam B., Şanlier N. (2017). Curcumin, an Active Component of Turmeric (*Curcuma longa*), and Its Effects on Health. Crit. Rev. Food Sci. Nutr..

[122] Kim J., Kim K., Jang J., Kim M.J., Cooray A., Lee Y., Lee K.P., Lee D.Y. (2025). Anti-Inflammatory and Antibacterial Properties of *Curcuma longa* Extract Against Helicobacter Pylori. Mol. Nutr. Food Res..

[123] Lee H.-Y., Kim S.-W., Lee G.-H., Choi M.-K., Jung H.-W., Kim Y.-J., Kwon H.-J., Chae H.-J. (2016). Turmeric Extract and Its Active Compound, Curcumin, Protect against Chronic CCl4-Induced Liver Damage by Enhancing Antioxidation. BMC Complement. Altern. Med..

[124] Essa R., El Sadek A.M., Baset M.E., Rawash M.A., Sami D.G., Badawy M.T., Mansour M.E., Attia H., Saadeldin M.K., Abdellatif A. (2019). Effects of Turmeric (*Curcuma longa*) Extract in Streptozocin-induced Diabetic Model. J. Food Biochem..

[125] Iweala E.J., Uche M.E., Dike E.D., Etumnu L.R., Dokunmu T.M., Oluwapelumi A.E., Okoro B.C., Dania O.E., Adebayo A.H., Ugbogu E.A. (2023). *Curcuma longa* (Turmeric): Ethnomedicinal Uses, Phytochemistry, Pharmacological Activities and Toxicity Profiles—A Review. Pharmacol. Res.-Mod. Chin. Med..

[126] Chen T.-C., Chuang J.-Y., Ko C.-Y., Kao T.-J., Yang P.-Y., Yu C.-H., Liu M.-S., Hu S.-L., Tsai Y.-T., Chan H. (2020). AR Ubiquitination Induced by the Curcumin Analog Suppresses Growth of Temozolomide-Resistant Glioblastoma through Disrupting GPX4-Mediated Redox Homeostasis. Redox Biol..

[127] Boddy L., Crockatt M.E., Ainsworth A.M. (2011). Ecology of *Hericium cirrhatum*, *H. coralloides* and *H. erinaceus* in the UK. Fungal Ecol..

[128] Chong P.S., Khairuddin S., Tse A.C.K., Hiew L.F., Lau C.L., Tipoe G.L., Fung M.-L., Wong K.H., Lim L.W. (2020). *Hericium erinaceus* Potentially Rescues Behavioural Motor Deficits through ERK-CREB-PSD95 Neuroprotective Mechanisms in Rat Model of 3-Acetylpyridine-Induced Cerebellar Ataxia. Sci. Rep..

[129] Chong P.S., Poon C.H., Roy J., Tsui K.C., Lew S.Y., Phang M.W.L., Tan R.J.Y., Cheng P.G., Fung M.-L., Wong K.H. (2021). Neurogenesis-Dependent Antidepressant-like Activity of *Hericium erinaceus* in an Animal Model of Depression. Chin. Med..

[130] Yanshree, Yu W.S., Fung M.L., Lee C.W., Lim L.W., Wong K.H. (2022). The Monkey Head Mushroom and Memory Enhancement in Alzheimer’s Disease. Cells.

[131] Contato A.G., Conte-Junior C.A. (2025). Lion’s Mane Mushroom (*Hericium erinaceus*): A Neuroprotective Fungus with Antioxidant, Anti-Inflammatory, and Antimicrobial Potential—A Narrative Review. Nutrients.

[132] Gravina A.G., Pellegrino R., Auletta S., Palladino G., Brandimarte G., D’Onofrio R., Arboretto G., Imperio G., Ventura A., Cipullo M. (2023). *Hericium erinaceus*, a Medicinal Fungus with a Centuries-Old History: Evidence in Gastrointestinal Diseases. World J. Gastroenterol..

[133] Zhong X., Wang G., Li F., Fang S., Zhou S., Ishiwata A., Tonevitsky A.G., Shkurnikov M., Cai H., Ding F. (2023). Immunomodulatory Effect and Biological Significance of β-Glucans. Pharmaceutics.

[134] Cao Y., Wang Q., Li L., Jiang H., Zhang B., Wu Y., Zhou F., Hua C., Huo G., Li S. (2025). Neuroprotective Terpenoids Derived from *Hericium erinaceus* Fruiting Bodies: Isolation, Structural Elucidation, and Mechanistic Insights. Int. J. Mol. Sci..

[135] Puia A., Pandrea S.-L., Cruceru J., Puia I.C., Chedea V.S., Ciont C., Pop O.L., Leopold L.F., Ranga F., Urcan A.C. (2025). Phenolic Compounds with Antimicrobial Properties in Mushrooms Frequently Encountered in Temperate Deciduous Forests. Life.

[136] Samberkar S., Gandhi S., Naidu M., Wong K.-H., Raman J., Sabaratnam V. (2015). Lion’s Mane, *Hericium erinaceus* and Tiger Milk, Lignosus Rhinocerotis (Higher Basidiomycetes) Medicinal Mushrooms Stimulate Neurite Outgrowth in Dissociated Cells of Brain, Spinal Cord, and Retina: An In Vitro Study. Int. J. Med. Mushrooms.

[137] Wong K.-H., Vikineswary S., Abdullah N., Naidu M., Keynes R. (2007). Activity of Aqueous Extracts of Lion’s Mane Mushroom *Hericium erinaceus* (Bull.: Fr.) Pers. (Aphyllophoromycetideae) on the Neural Cell Line NG108-15. Int. J. Med. Mushrooms.

[138] Wong K.-H., Naidu M., David P., Abdulla M.A., Abdullah N., Kuppusamy U.R., Sabaratnam V. (2011). Peripheral Nerve Regeneration Following Crush Injury to Rat Peroneal Nerve by Aqueous Extract of Medicinal Mushroom *Hericium erinaceus* (Bull.: Fr) Pers. (Aphyllophoromycetideae). Evid.-Based Complement. Altern. Med..

[139] Wong K.-H., Kanagasabapathy G., Naidu M., David P., Sabaratnam V. (2016). *Hericium erinaceus* (Bull.: Fr.) Pers., a Medicinal Mushroom, Activates Peripheral Nerve Regeneration. Chin. J. Integr. Med..

[140] Cordaro M., Salinaro A.T., Siracusa R., D’Amico R., Impellizzeri D., Scuto M., Ontario M.L., Cuzzocrea S., Di Paola R., Fusco R. (2021). Key Mechanisms and Potential Implications of *Hericium erinaceus* in NLRP3 Inflammasome Activation by Reactive Oxygen Species during Alzheimer’s Disease. Antioxidants.

[141] Priori E.C., Ratto D., De Luca F., Sandionigi A., Savino E., Giammello F., Romeo M., Brandalise F., Roda E., Rossi P. (2024). *Hericium erinaceus* Extract Exerts Beneficial Effects on Gut–Neuroinflammaging–Cognitive Axis in Elderly Mice. Biology.

[142] Hsu P.-C., Lan Y.-J., Chen C.-C., Lee L.-Y., Chen W.-P., Wang Y.-C., Lee Y.-H. (2022). Erinacine A Attenuates Glutamate Transporter 1 Downregulation and Protects against Ischemic Brain Injury. Life Sci..

[143] Hu P., Suriguga, Zhao M., Chen S., Wu X., Wan Q. (2022). Transcriptional Regulation Mechanism of Flavonoids Biosynthesis Gene during Fruit Development in *Astragalus membranaceus*. Front. Genet..

[144] Li X., Qu L., Dong Y., Han L., Liu E., Fang S., Zhang Y., Wang T. (2014). A Review of Recent Research Progress on the Astragalus Genus. Molecules.

[145] Sevgi S., Cavusoglu Nalbantoglu I., Kerimoglu G., Kesim S.M., Burnett A.L., Sezen S.F. (2026). *Astragalus membranaceus* Fisch. Ex Bunge-Derived Astragaloside IV Recovers Bilateral Cavernous Nerve Injury-Induced Erectile Dysfunction and Corporal Fibrosis by Inhibiting the TGF-Β1/Smad2 Pathway in Rats. J. Ethnopharmacol..

[146] Cui Z., Shang Q. (2025). Mechanistic Insights into the Antitumor Effects of Astragaloside IV and Astragalus Polysaccharide in Digestive System Cancers. Front. Pharmacol..

[147] Zhang Q.-L., Qin W.-X., Li X.-J., Zhang Y.-B., Li M., Xu J.-F., Mao Z.-N. (2026). Astragaloside IV Is a Potential Natural Neuroprotective Agent for Stroke: A Review. Front. Pharmacol..

[148] Liu P., Zhao H., Luo Y. (2017). Anti-Aging Implications of *Astragalus membranaceus* (Huangqi): A Well-Known Chinese Tonic. Aging Dis..

[149] Lu M.-C., Yao C.-H., Wang S.-H., Lai Y.-L., Tsai C.-C., Chen Y.-S. (2010). Effect of *Astragalus membranaceus* in Rats on Peripheral Nerve Regeneration: In Vitro and in Vivo Studies. J. Trauma.

[150] Liang Y., Chen B., Liang D., Quan X., Gu R., Meng Z., Gan H., Wu Z., Sun Y., Liu S. (2023). Pharmacological Effects of Astragaloside IV: A Review. Molecules.

[151] Jia Y., Wang Y., Wang Z., Zhang Z., Zhang J., Zhang J., Sun K., Hua Y., Chai G., Hu F. (2024). Neuroprotective Effects of Total Phenolics from *Hemerocallis citrina* Baroni Leaves through the PI3K/AKT Pathway. Front. Pharmacol..

[152] Gu L., Liu Y.-J., Wang Y.-B., Yi L.-T. (2012). Role for Monoaminergic Systems in the Antidepressant-like Effect of Ethanol Extracts from *Hemerocallis citrina*. J. Ethnopharmacol..

[153] Ma T., Sun Y., Wang L., Wang J., Wu B., Yan T., Jia Y. (2022). An Investigation of the Anti-Depressive Properties of Phenylpropanoids and Flavonoids in *Hemerocallis citrina* Baroni. Molecules.

[154] Sun Y., Fu C., Bu H., Zhang J., Ma Y., Hong Y., Wang W., Bo S., Ma T. (2025). Anti-Inflammatory Constituents from *Hemerocallis citrina* Roots. Chem. Nat. Compd..

[155] Zou Y., Qin X., Wang W., Meng Q., Zhang Y. (2024). Anti-Aging Effect of *Hemerocallis citrina* Baroni Polysaccharide-Rich Extract on *Caenorhabditis elegans*. Int. J. Mol. Sci..

[156] Khan A., Park J.S., Kang M.H., Lee H.J., Ali J., Tahir M., Choe K., Kim M.O. (2023). Caffeic Acid, a Polyphenolic Micronutrient Rescues Mice Brains against Aβ-Induced Neurodegeneration and Memory Impairment. Antioxidants.

[157] Kowalczyk A., Tuberoso C.I.G., Jerković I. (2025). Experimental Evidence of Caffeic Acid’s Neuroprotective Activity in Alzheimer’s Disease: In Vitro, In Vivo, and Delivery-Based Insights. Medicina.

[158] Saenno R., Dornlakorn O., Anosri T., Kaewngam S., Sirichoat A., Aranarochana A., Pannangrong W., Wigmore P., Welbat J.U. (2022). Caffeic Acid Alleviates Memory and Hippocampal Neurogenesis Deficits in Aging Rats Induced by D-Galactose. Nutrients.

[159] Pavlíková N. (2022). Caffeic Acid and Diseases—Mechanisms of Action. Int. J. Mol. Sci..

[160] Kamarauskaite J., Baniene R., Trumbeckas D., Strazdauskas A., Trumbeckaite S. (2021). Caffeic Acid Phenethyl Ester Protects Kidney Mitochondria against Ischemia/Reperfusion Induced Injury in an In Vivo Rat Model. Antioxidants.

[161] Meng T., Xiao D., Muhammed A., Deng J., Chen L., He J. (2021). Anti-Inflammatory Action and Mechanisms of Resveratrol. Molecules.

[162] Wang Y., Li Y., He C., Gou B., Song M. (2019). Mitochondrial Regulation of Cardiac Aging. Biochim. Biophys. Acta (BBA) -Mol. Basis Dis..

[163] Arafa M.H., Mohammad N.S., Atteia H.H., Abd-Elaziz H.R. (2014). Protective Effect of Resveratrol against Doxorubicin-Induced Cardiac Toxicity and Fibrosis in Male Experimental Rats. J. Physiol. Biochem..

[164] Nunes S., Danesi F., Rio D.D., Silva P. (2018). Resveratrol and Inflammatory Bowel Disease: The Evidence so Far. Nutr. Res. Rev..

[165] Alarcón de la Lastra C., Villegas I. (2005). Resveratrol as an Anti-Inflammatory and Anti-Aging Agent: Mechanisms and Clinical Implications. Mol. Nutr. Food Res..

[166] Lançon A., Delma D., Osman H., Thénot J.-P., Latruffe B.J. (2004). Human Hepatic Cell Uptake of Resveratrol: Involvement of Both Passive Diffusion and Carrier-Mediated Process. Biochem. Biophys. Res. Commun..

[167] Baur J.A., Pearson K.J., Price N.L., Jamieson H.A., Lerin C., Kalra A., Prabhu V.V., Allard J.S., Lopez-Lluch G., Lewis K. (2006). Resveratrol Improves Health and Survival of Mice on a High-Calorie Diet. Nature.

[168] Goncalves R.L.S., Quinlan C.L., Perevoshchikova I.V., Hey-Mogensen M., Brand M.D. (2015). Sites of Superoxide and Hydrogen Peroxide Production by Muscle Mitochondria Assessed Ex Vivo under Conditions Mimicking Rest and Exercise. J. Biol. Chem..

[169] Kowalczyk P., Sulejczak D., Kleczkowska P., Bukowska-Ośko I., Kucia M., Popiel M., Wietrak E., Kramkowski K., Wrzosek K., Kaczyńska K. (2021). Mitochondrial Oxidative Stress—A Causative Factor and Therapeutic Target in Many Diseases. Int. J. Mol. Sci..

[170] Liao X., Han Y., He Y., Liu J., Wang Y. (2023). Natural Compounds Targeting Mitochondrial Dysfunction: Emerging Therapeutics for Target Organ Damage in Hypertension. Front. Pharmacol..

[171] Cherry A.D., Suliman H.B., Bartz R.R., Piantadosi C.A. (2014). Peroxisome Proliferator-Activated Receptor γ Co-Activator 1-α as a Critical Co-Activator of the Murine Hepatic Oxidative Stress Response and Mitochondrial Biogenesis in *Staphylococcus aureus* Sepsis. J. Biol. Chem..

[172] Lin J., Wu P.-H., Tarr P.T., Lindenberg K.S., St-Pierre J., Zhang C., Mootha V.K., Jäger S., Vianna C.R., Reznick R.M. (2004). Defects in Adaptive Energy Metabolism with CNS-Linked Hyperactivity in PGC-1α Null Mice. Cell.

[173] Kwik M., Hainzl S., Oppelt J., Tichy B., Koller U., Bernardinelli E., Steiner M., Zara G., Nofziger C., Weis S. (2021). Selective Activation of CNS and Reference PPARGC1A Promoters Is Associated with Distinct Gene Programs Relevant for Neurodegenerative Diseases. Int. J. Mol. Sci..

[174] Khan T., Waseem R., Zehra Z., Aiman A., Bhardwaj P., Ansari J., Hassan M.I., Islam A. (2022). Mitochondrial Dysfunction: Pathophysiology and Mitochondria-Targeted Drug Delivery Approaches. Pharmaceutics.

[175] Wen H., Deng H., Li B., Chen J., Zhu J., Zhang X., Yoshida S., Zhou Y. (2025). Mitochondrial diseases: From molecular mechanisms to therapeutic advances. Signal Transduct. Target. Ther..

[176] Kostanda E., Musa S., Pereman I. (2024). Unveiling the Chemical Composition and Biofunctionality of Hericium Spp. Fungi: A Comprehensive Overview. Int. J. Mol. Sci..

[177] Urbonaviciene D., Bobinaite R., Viskelis P., Bobinas C., Petruskevicius A., Klavins L., Viskelis J. (2022). Geographic Variability of Biologically Active Compounds, Antioxidant Activity and Physico-Chemical Properties in Wild Bilberries (*Vaccinium myrtillus* L.). Antioxidants.

[178] Xiong H., Yu L.X., Qu H. (2013). Batch-to-Batch Quality Consistency Evaluation of Botanical Drug Products Using Multivariate Statistical Analysis of the Chromatographic Fingerprint. AAPS PharmSciTech.

[179] Kała K., Cicha-Jeleń M., Sułkowska-Ziaja K., Ostachowicz B., Węgrzynowicz E., Lazur J., Szewczyk A., Muszyńska B. (2025). Influence of Plant-Based Substrate Composition and Extraction Method on Accumulation of Bioactive Compounds in *Hericium erinaceus* (Bull.) Pers. Fruiting Bodies. Molecules.

[180] Ryu S.H., Kim B.S., Kim S.J., Kang D.-M., Lee H.H., Yeon S.W., Ahn M.-J., Hwang B.Y., Lee M.K. (2026). Nutritional and Functional Enhancement of Lion’s Mane Mushroom (*Hericium erinaceus*) via Sustainable Brown Rice Cultivation. Future Foods.

[181] Zheng L., Wang M., Ibarra-Estrada E., Wu C., Wilson E.G., Verpoorte R., Klinkhamer P.G.L., Choi Y.H. (2015). Investigation of Chemomarkers of Astragali Radix of Different Ages and Geographical Origin by NMR Profiling. Molecules.

[182] Muyumba N.W., Mutombo S.C., Sheridan H., Nachtergael A., Duez P. (2021). Quality Control of Herbal Drugs and Preparations: The Methods of Analysis, Their Relevance and Applications. Talanta Open.

[183] Kanubaddi K.R., Yaung C.-L., Harn H.-J., Chiou T.-W., Hsu S.-X., Wijaya I., Lin S.-Z., Wuli W. (2026). Astragalus and Cordyceps Derivatives in the Treatment of Aging-Related Chronic Diseases and Neurodegenerative Disorders. Int. J. Mol. Sci..

[184] Wang H., Chen Y., Wang L., Liu Q., Yang S., Wang C. (2023). Advancing Herbal Medicine: Enhancing Product Quality and Safety through Robust Quality Control Practices. Front. Pharmacol..

[185] Noviana E., Indrayanto G., Rohman A. (2022). Advances in Fingerprint Analysis for Standardization and Quality Control of Herbal Medicines. Front. Pharmacol..

[186] Nasim N., Sandeep I.S., Mohanty S. (2022). Plant-Derived Natural Products for Drug Discovery: Current Approaches and Prospects. Nucleus.

[187] Kaushik R., Jain J., Mazumder A. (2018). Chromatographic Fingerprinting of Sarasvata Churna-an Ayurvedic Polyherbal Formulation for Epilepsy. japs.

[188] Cordeiro C.F., Franco L.L., Carvalho D.T., Bonfilio R. (2024). Impurities in Active Pharmaceutical Ingredients and Drug Products: A Critical Review. Crit. Rev. Anal. Chem..

[189] Ma Y., Zhao Y., Luo M., Jiang Q., Liu S., Jia Q., Bai Z., Wu F., Xie J. (2024). Advancements and Challenges in Pharmacokinetic and Pharmacodynamic Research on the Traditional Chinese Medicine Saponins: A Comprehensive Review. Front. Pharmacol..

[190] Mohapatra D., Agrawal A.K., Sahu A.N. (2021). Exploring the Potential of Solid Dispersion for Improving Solubility, Dissolution & Bioavailability of Herbal Extracts, Enriched Fractions, and Bioactives. J. Microencapsul..

[191] Paroha S., Dewangan R.P., Sahoo P.K., Saneja A., Panda A.K., Lichtfouse E. (2020). Pharmaceutical Technology for Improving the Bioavailability of Natural Products. Sustainable Agriculture Reviews 43: Pharmaceutical Technology for Natural Products Delivery Vol. 1 Fundamentals and Applications.

[192] Bhalani D.V., Nutan B., Kumar A., Singh Chandel A.K. (2022). Bioavailability Enhancement Techniques for Poorly Aqueous Soluble Drugs and Therapeutics. Biomedicines.

[193] Kesarwani K., Gupta R. (2013). Bioavailability Enhancers of Herbal Origin: An Overview. Asian Pac. J. Trop. BioMed..

[194] Lestari U., Muhaimin M., Chaerunisaa A.Y., Sujarwo W. (2023). Improved Solubility and Activity of Natural Product in Nanohydrogel. Pharmaceuticals.

[195] Cosmetic Ingredient Review Expert Panel (2007). Final report on the safety assessment of glycyrrhetinic acid, potassium glycyrrhetinate, disodium succinoyl glycyrrhetinate, glyceryl glycyrrhetinate, glycyrrhetinyl stearate, stearyl glycyrrhetinate, glycyrrhizic acid, ammonium glycyrrhizate, dipotassium glycyrrhizate, disodium glycyrrhizate, trisodium glycyrrhizate, methyl glycyrrhizate, and potassium glycyrrhizinate. Int. J. Toxicol..

[196] Weng Q., Chen L., Ye L., Lu X., Yu Z., Wen C., Chen Y., Huang G. (2019). Determination of Licochalcone A in Rat Plasma by UPLC–MS/MS and Its Pharmacokinetics. Acta Chromatogr..

[197] Genchi G., Lauria G., Catalano A., Carocci A., Sinicropi M.S. (2024). Neuroprotective Effects of Curcumin in Neurodegenerative Diseases. Foods.

[198] Prasad S., Tyagi A.K., Aggarwal B.B. (2014). Recent Developments in Delivery, Bioavailability, Absorption and Metabolism of Curcumin: The Golden Pigment from Golden Spice. Cancer Res. Treat..

[199] Tsai P.-C., Wu Y.-K., Hu J.-H., Li I.-C., Lin T.-W., Chen C.-C., Kuo C.-F. (2021). Preclinical Bioavailability, Tissue Distribution, and Protein Binding Studies of Erinacine A, a Bioactive Compound from *Hericium erinaceus* Mycelia Using Validated LC-MS/MS Method. Molecules.

[200] Gu Y., Wang G., Pan G., Fawcett J.P., A J., Sun J. (2004). Transport and Bioavailability Studies of Astragaloside IV, an Active Ingredient in *Radix Astragali*. Basic Clin. Pharmacol. Toxicol..

[201] Huang C.R., Wang G.J., Wu X.L., Li H., Xie H.T., Lv H., Sun J.G. (2006). Absorption Enhancement Study of Astragaloside IV Based on Its Transport Mechanism in Caco-2 Cells. Eur. J. Drug Metab. Pharmacokinet..

[202] Zhang L. (2019). Pharmacokinetics and Drug Delivery Systems for Puerarin, a Bioactive Flavone from Traditional Chinese Medicine. Drug Deliv..

[203] Ahmad S., Ahsan F., Ansari J.A., Mahmood T., Shamim A., Bano S., Tiwari R., Ansari V.A., Shafiurrahman, Kesari M. (2024). A Review on Daidzein as Food Supplement: Exploring its Phytopharmacological and Preclinical Status. eFood.

[204] Laurindo L.F., Rodrigues V.D., Guiguer E.L., Laurindo L.F., de Campos Zuccari D.A.P., Detregiachi C.R.P., Araújo A.C., da Silva Camarinha Oliveira J., Maria D.A., Dias J.A. (2025). Catalpol: An Iridoid Glycoside with Potential in Combating Cancer Development and Progression-A Comprehensive Review. Phytother. Res..

[205] Wang S.-J., Zeng J., Yang B.-K., Zhong Y.-M. (2014). Bioavailability of Caffeic Acid in Rats and Its Absorption Properties in the Caco-2 Cell Model. Pharm. Biol..

[206] Böhmdorfer M., Szakmary A., Schiestl R.H., Vaquero J., Riha J., Brenner S., Thalhammer T., Szekeres T., Jäger W. (2017). Involvement of UDP-Glucuronosyltransferases and Sulfotransferases in the Excretion and Tissue Distribution of Resveratrol in Mice. Nutrients.

[207] Ndiaye M., Kumar R., Ahmad N. (2011). Resveratrol in Cancer Management: Where Are We and Where We Go from Here?. Ann. N. Y. Acad. Sci..

[208] Chiu C., Cheng W., Lin T., Chang H., Tseng M., Chang Y., Su S., Lee C., Chang H., Liu C. (2026). *Astragalus membranaceus* Therapy in Patients with Spinocerebellar Ataxia Type 3. J. Tradit. Complement. Med..

[209] Moise G., Jîjie A.-R., Moacă E.-A., Predescu I.-A., Dehelean C.A., Hegheș A., Vlad D.C., Popescu R., Vlad C.S. (2024). Plants’ Impact on the Human Brain—Exploring the Neuroprotective and Neurotoxic Potential of Plants. Pharmaceuticals.

[210] Zhang J., Onakpoya I.J., Posadzki P., Eddouks M. (2015). The Safety of Herbal Medicine: From Prejudice to Evidence. Evid. Based Complement. Altern. Med..

[211] Guo W., Xing Y., Luo X., Li F., Ren M., Liang Y. (2023). Reactive Oxygen Species: A Crosslink between Plant and Human Eukaryotic Cell Systems. Int. J. Mol. Sci..

[212] Marie O.M., Abu-Elsaoud A. (2026). Obesity and Natural Products: Mechanisms, Therapeutic Potential, and Future Directions. Food Sci. Nutr..

[213] Ekor M. (2014). The Growing Use of Herbal Medicines: Issues Relating to Adverse Reactions and Challenges in Monitoring Safety. Front. Pharmacol..

[214] Husain I., Bala K., Khan I.A., Khan S.I. (2021). A Review on Phytochemicals, Pharmacological Activities, Drug Interactions, and Associated Toxicities of Licorice (*Glycyrrhiza* Sp.). Food Front..

[215] Eid H.M., Vallerand D., Muhammad A., Durst T., Haddad P.S., Martineau L.C. (2010). Structural Constraints and the Importance of Lipophilicity for the Mitochondrial Uncoupling Activity of Naturally Occurring Caffeic Acid Esters with Potential for the Treatment of Insulin Resistance. Biochem. Pharmacol..

[216] Caesar L.K., Cech N.B. (2019). Synergy and Antagonism in Natural Product Extracts: When 1 + 1 Does Not Equal 2. Nat. Prod. Rep..

[217] John P.A., Wong K.-H., Naidu M., Sabaratnam V., David P. (2013). Combination Effects of Curcumin and Aqueous Extract of Lignosus Rhinocerotis Mycelium on Neurite Outgrowth Stimulation Activity in PC-12 Cells. Nat. Product. Commun..

[218] Pemovska T., Bigenzahn J.W., Superti-Furga G. (2018). Recent Advances in Combinatorial Drug Screening and Synergy Scoring. Curr. Opin. Pharmacol..

[219] Yuan H., Ma Q., Cui H., Liu G., Zhao X., Li W., Piao G. (2017). How Can Synergism of Traditional Medicines Benefit from Network Pharmacology?. Molecules.

[220] Joyce N., Annett G., Wirthlin L., Olson S., Bauer G., Nolta J.A. (2010). Mesenchymal Stem Cells for the Treatment of Neurodegenerative Disease. Regen. Med..

[221] Corcione A., Benvenuto F., Ferretti E., Giunti D., Cappiello V., Cazzanti F., Risso M., Gualandi F., Mancardi G.L., Pistoia V. (2006). Human Mesenchymal Stem Cells Modulate B-Cell Functions. Blood.

[222] Jones B.J., Brooke G., Atkinson K., McTaggart S.J. (2007). Immunosuppression by Placental Indoleamine 2,3-Dioxygenase: A Role for Mesenchymal Stem Cells. Placenta.

[223] Seo J.H., Cho S.-R. (2012). Neurorestoration Induced by Mesenchymal Stem Cells: Potential Therapeutic Mechanisms for Clinical Trials. Yonsei Med. J..

[224] Skok M. (2021). Mesenchymal Stem Cells as a Potential Therapeutic Tool to Cure Cognitive Impairment Caused by Neuroinflammation. World J. Stem Cells.

[225] Wu T., Liu Y., Wang S., Shi C. (2025). MSC-Derived Extracellular Vesicles: Roles and Molecular Mechanisms for Tissue Repair. Int. J. Nanomed..

[226] Yin K., Wang S., Zhao R.C. (2019). Exosomes from Mesenchymal Stem/Stromal Cells: A New Therapeutic Paradigm. Biomark. Res..

[227] Chang Y.-K., Chen M.-H., Chiang Y.-H., Chen Y.-F., Ma W.-H., Tseng C.-Y., Soong B.-W., Ho J.H., Lee O.K. (2011). Mesenchymal Stem Cell Transplantation Ameliorates Motor Function Deterioration of Spinocerebellar Ataxia by Rescuing Cerebellar Purkinje Cells. J. BioMed. Sci..

[228] Lee O.K., Kuo T.K., Chen W.-M., Lee K.-D., Hsieh S.-L., Chen T.-H. (2004). Isolation of Multipotent Mesenchymal Stem Cells from Umbilical Cord Blood. Blood.

[229] Wu J., Ge Y., Huang W., Zhang L., Huang J., Huang N., Luo Y. (2025). Natural Bioactive Compounds Modified with Mesenchymal Stem Cells: New Hope for Regenerative Medicine. Front. Bioeng. Biotechnol..

[230] Tseng P., Hou S., Chen R., Peng H., Hsieh C., Kuo M., Yen M. (2011). Resveratrol Promotes Osteogenesis of Human Mesenchymal Stem Cells by Upregulating RUNX2 Gene Expression via the SIRT1/FOXO3A Axis. J. Bone Min. Res..

[231] Patel M.M., Patel B.M. (2017). Crossing the Blood–Brain Barrier: Recent Advances in Drug Delivery to the Brain. CNS Drugs.

[232] Pardridge W.M. (2005). The Blood-Brain Barrier: Bottleneck in Brain Drug Development. NeuroRx.

[233] Da Silva J.D., Teixeira-Castro A., Maciel P. (2019). From Pathogenesis to Novel Therapeutics for Spinocerebellar Ataxia Type 3: Evading Potholes on the Way to Translation. Neurotherapeutics.

[234] Sanjai C., Gaonkar S.L., Hakkimane S.S. (2024). Harnessing Nature’s Toolbox: Naturally Derived Bioactive Compounds in Nanotechnology Enhanced Formulations. ACS Omega.

[235] Hersh A.M., Alomari S., Tyler B.M. (2022). Crossing the Blood-Brain Barrier: Advances in Nanoparticle Technology for Drug Delivery in Neuro-Oncology. Int. J. Mol. Sci..

[236] Desai N., Rana D., Patel M., Bajwa N., Prasad R., Vora L.K. (2025). Nanoparticle Therapeutics in Clinical Perspective: Classification, Marketed Products, and Regulatory Landscape. Small.

[237] Griffin S., Masood M.I., Nasim M.J., Sarfraz M., Ebokaiwe A.P., Schäfer K.-H., Keck C.M., Jacob C. (2017). Natural Nanoparticles: A Particular Matter Inspired by Nature. Antioxidants.

[238] Joudeh N., Linke D. (2022). Nanoparticle Classification, Physicochemical Properties, Characterization, and Applications: A Comprehensive Review for Biologists. J. Nanobiotechnol..

[239] Ashfaq R., Rasul A., Asghar S., Kovács A., Berkó S., Budai-Szűcs M. (2023). Lipid Nanoparticles: An Effective Tool to Improve the Bioavailability of Nutraceuticals. Int. J. Mol. Sci..

[240] Geszke-Moritz M., Moritz M. (2024). Biodegradable Polymeric Nanoparticle-Based Drug Delivery Systems: Comprehensive Overview, Perspectives and Challenges. Polymers.

[241] Lamade A.M., Kenny E.M., Anthonymuthu T.S., Soysal E., Clark R., Kagan V.E., Bayir H. (2019). Aiming for the Target: Mitochondrial Drug Delivery in Traumatic Brain Injury. Neuropharmacology.

[242] Zhang X., Chen J., Wan B., Zheng Y., Chen X. (2025). Mitochondria-Targeted Nanosystems in the Treatment of Central Nervous System Diseases. Int. J. Nanomed..

[243] Kurt S., Kimiz-Gebologlu I., Cinar O., Oncel S.S. (2026). Natural Nanocarriers in Oncology: The Targeting and Delivery Capabilities of Extracellular Vesicles. OpenNano.

[244] Faouzi A., Roullin V.G. (2021). Think Big, Start Small: How Nanomedicine Could Alleviate the Burden of Rare CNS Diseases. Pharmaceuticals.

